# Molecular Evolution of Extensively Drug-Resistant (XDR) *Pseudomonas aeruginosa* Strains From Patients and Hospital Environment in a Prolonged Outbreak

**DOI:** 10.3389/fmicb.2019.01742

**Published:** 2019-08-08

**Authors:** Michael Buhl, Christina Kästle, André Geyer, Ingo B. Autenrieth, Silke Peter, Matthias Willmann

**Affiliations:** ^1^Institute of Medical Microbiology and Hygiene, University of Tübingen, Tübingen, Germany; ^2^German Center for Infection Research, Partner Site Tübingen, Tübingen, Germany

**Keywords:** extensive drug resistance, single-nucleotide polymorphisms, SNPs, protein enrichment, functional enrichment, mercury, whole genome sequencing, WGS

## Abstract

In this study, we aimed to elucidate a prolonged outbreak of extensively drug-resistant (XDR) *Pseudomonas aeruginosa*, at two adjacent hospitals over a time course of 4 years. Since all strains exhibited a similar antibiotic susceptibility pattern and carried the carbapenemase gene bla_VIM_, a monoclonal outbreak was assumed. To shed light on the intra-hospital evolution of these strains over time, whole genome sequence (WGS) analysis of 100 clinical and environmental outbreak strains was employed. Phylogenetic analysis of the core genome revealed the outbreak to be polyclonal, rather than monoclonal as initially suggested. The vast majority of strains fell into one of two major clusters, composed of 27 and 59 strains, and their accessory genome each revealed over 400 and 600 accessory genes, respectively, thus indicating an unexpectedly high structural diversity among phylogenetically clustered strains. Further analyses focused on the cluster with 59 strains, representing the hospital from which both clinical and environmental strains were available. Our investigation clearly shows both accumulation and loss of genes occur very frequently over time, as reflected by analysis of protein enrichment as well as functional enrichment. In addition, we investigated adaptation through single nucleotide polymorphisms (SNPs). Among the genes affected by SNPs, there are a multidrug efflux pump (*mexZ*) and a mercury detoxification operon (*merR*) with deleterious mutations, potentially leading to loss of repression with resistance against antibiotics and disinfectants. Our results not only confirm WGS to be a powerful tool for epidemiologic analyses, but also provide insights into molecular evolution during an XDR *P. aeruginosa* hospital outbreak. Genome mutation unveiled a striking genetic plasticity on an unexpectedly high level, mostly driven by horizontal gene transfer. Our study adds valuable information to the molecular understanding of “real-world” Intra-hospital *P. aeruginosa* evolution and is a step forward toward more personalized medicine in infection control.

## Introduction

*Pseudomonas aeruginosa* (PA) is a non-fermentative Gram-negative bacterium commonly found in wet environments. It is recognized as an important human pathogen known to especially affect patients at risk with conditions such as immunosuppression, burn wounds and cystic fibrosis (CF) (Azzopardi et al., 2014; Tatarelli and Mikulska, 2016; Stefani et al., 2017). In the nosocomial context, particularly multi- or extensively drug-resistant (MDR, XDR) strains pose a global threat to vulnerable patients and healthcare systems and have been reported to cause numerous hospital outbreaks involving high-risk patients such as in intensive care units (ICUs) or hemato-oncology wards (Buhl et al., 2015).

In this study, we analyzed a prolonged outbreak of XDR *P. aeruginosa* (XDR-PA) involving two adjacent hospitals, namely a 330-bed casualty hospital (hospital A) and a 1,500-bed teaching hospital (hospital B) in Germany. The XDR-PA strains were routinely sampled from patients (colonization and infection) and from the hospital environment (including toilets and washing basins).

All strains had a similar antibiotic susceptibility pattern and carried the bla_VIM_ gene. For these reasons, we suspected a monoclonal outbreak. Our primary study objectives were (i) a detailed strain typing using whole genome sequencing (WGS) in order to confirm our initial suspicion of a monoclonal outbreak, and (ii) a molecular evolution analysis of outbreak strains over a time course of 4 years. The latter point is particularly important. During the outbreak, infection prevention and control (IPC) measures were intensified. Despite these interventions, we have continuously isolated XDR-PA strains from patients and found them in the hospital environment where they most likely persisted; a relevant but often overlooked mode of transmission (De Abreu et al., 2014).

It is tempting to ask whether genomic variations promoted an adaptation to hospital environment and IPC measures. Molecular evolution of long-term outbreak strains is likely driven by adaptive mechanisms in response to IPC measures (e.g., disinfection of sanitary regions/rooms, application of tube cleaners). For example, a recent investigation of resistance development against alcohol-based disinfectants observed over time in VRE (vancomycin-resistant enterococci) has revealed molecular evidence to be the underlying cause (Pidot et al., 2018). WGS has also been successfully applied to investigate the molecular evolution of *Pseudomonas aeruginosa* in cystic fibrosis (CF) patients where it allowed for unprecedented insights (Marvig et al., 2015). Such a molecular understanding of bacterial evolution is not only of great interest in CF patients, but also in immunocompromised patients susceptible to opportunistic infections and with potentially greater transmission rates especially in the nosocomial context. Molecular data elucidating genes related to environmental stress resistance may provide hints about the effectiveness of IPC measures especially in the context of hospital outbreaks and beyond. These data may not only provide clues as for implementation of targeted approaches, e.g., in case of resistance against alcohol-based disinfectants, but may also provide an interesting starting point by identifying potential novel targets for future development of active agents or other strategies to combat Intra-hospital persistence (Juhas, 2015).

## Results

### Core Genome Phylogeny and Epidemiological Links

**Figure 1:**
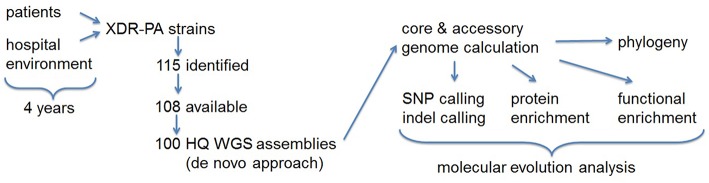
Project workflow summary flowchart. XDR-PA, extensively drug-resistant *Pseudomonas aeruginosa*; HQ, high quality; WGS, whole genome sequence; SNP, single nucleotide polymorphism.

We have sequenced 100 presumably monoclonal XDR-PA outbreak strains from two hospitals isolated over a time course of 4 years (Figure 1). Their core genome, representing the genetic backbone which all strains have in common, was calculated to be 5,508,057 base pairs in length, with a total number of 94,696 (1.7%) single nucleotide polymorphisms (SNPs), thereof 81,247 (85.8%) parsim-informatives and 13,449 (14.2%) singletons. This reflected a much higher rate of structural variations than expected from a monoclonal outbreak.

**Figure 2:**
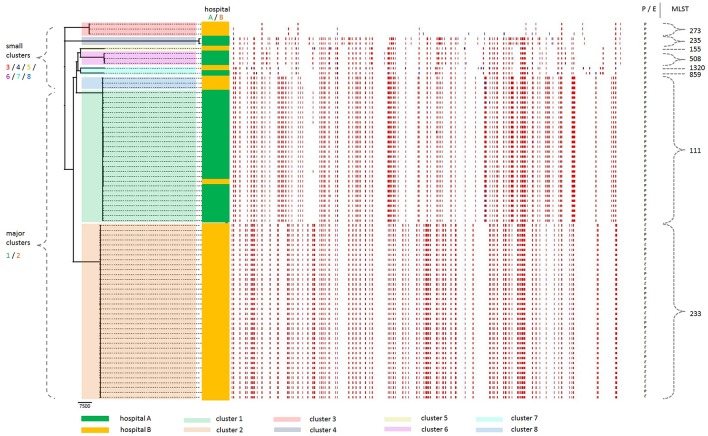
Core genome phylogeny of the 100 XDR *P. aeruginosa* outbreak strains included in this study, taking recombinational events into account. Out of these 100 strains, 23 strains (all from cluster 2) were excluded by the Gubbins algorithm because they were genetically identical with one of the other strains. Thus, only 36 strains of cluster 2 (constituted of a total of 59 strains) are shown. Grouping into clusters 1–8 is indicated in translucent colors: (i) two major clusters 1 and 2 (green and orange, respectively), and (ii) six small clusters 3, 4, 5, 6, 7, and 8 (red, gray, yellow, violet, turquoise, and blue, respectively). Sampling source (hospital A and B) is indicated for each strain in non-translucent colors (dark green and dark orange, respectively). As evident from the branches in the *far left of the figure*, the phylogenetic tree shows two major clusters, designated clusters 1 and 2, alongside with the six additional small clusters, designated cluster 3–8. In the *middle left of the figure*, the color coding (green/orange) indicates in which of the two hospitals (hospital A/B, respectively) the individual strains were sampled. In the *middle* and to the *right of the figure*, the colored barcode lines (red and blue) represent predicted recombinations, either shared by multiple isolates through common descent (red blocks), or occurring on terminal branches which are unique to individual isolates (blue blocks). In the *far right of the figure*, the source of the strains [P, patient (bold) or E, environment (italic)] and the MLST type are indicated. MLST, multi-locus sequence typing; XDR, extensively drug-resistant.

A core genome maximum-likelihood phylogenetic analysis revealed grouping into two major clusters (numbered 1 and 2) plus six small clusters (3–8) (Figure 2). From the 100 strains, 86 fall either into cluster 1 (*n* = 27) or cluster 2 (*n* = 59), and the remaining 14 strains are distributed over the small clusters 3–8. Where the outbreak had been assumed to be monoclonal because all strains exhibited an XDR phenotype with a similar antibiotic susceptibility pattern and carried the carbapenemase gene bla_VIM_, this data instead revealed a polyclonal outbreak setting with two major clusters.

Correlating this phylogenetic distribution with the source of the strains, it becomes evident that the two major clusters largely correspond to the two hospitals. In cluster 1, all but one of the 27 strains were sampled from hospital A. In cluster 2, all 59 strains were sampled from hospital B. Thus, the phylogeny reflects the sampling source of the strains from either of the two hospitals. The 100 XDR-PA strains investigated in this study are comprised of both clinical and environmental samples. Clinical samples were obtained from a total of 23 individual patients and originated from various sampling sites (colonization and infection), including e.g., rectal screening swabs as well as blood cultures. Different total numbers of *P. aeruginosa* strains per patient were included in this study, with a range from 1 to 21 strains per patient and an average of 3.1 strains per patient (Supplementary Table 1). In addition to clinical strains sampled from hospital A and B, environmental strains sampled from hospital B (but not from hospital A) were also included in this study. Environmental samples originated mostly from toilets and washing basins but also from, e.g., the toilet cistern and the corresponding inlet or outlet.

In cluster 1, all 27 strains are clinical samples (corresponding to 3 individual patients). Thereof, only one strain is from hospital B (sampled from patient 1), whereas all the other strains are from hospital A (patient 2 and 3). In cluster 2, the 59 strains include 31 clinical samples (corresponding to 15 individual patients) and 28 environmental samples. Within cluster 2, 36 strains were calculated to be genetically unique (these are shown in Figure 2), whereas the remaining 23 strains were genetically identical with one of the other strains (Supplementary Table 2). Taken together, cluster 2 includes more strains and represents a wider spectrum of sampling than cluster 1. Therefore, cluster 1 is less suitable for in-hospital molecular evolution analysis, for which exchange and interaction between clinical and environmental strains is assumed to be essential. This study focuses on a molecular evolution analysis of cluster 2, which includes both clinical and environmental strains.

In cluster 1, all but one strain had been sampled from two patients, namely patient 2 (*n* = 6) and patient 3 (*n* = 20). Taking into account the timeline of occurrence of these strains, there is no temporal overlap between these two patients, as the last XDR-PA strain from patient 2 (strain ID 66, day 501) had been sampled ~2 months before the first strain from patient 3 (strain ID 10, day 565) (Supplementary Table 3a). However, there is a spatial overlap for these two patients who had been admitted to the same ICU at hospital A. This suggests either an environmental source within hospital A where the strain was transmitted to the patients, or an independent introduction into the hospital by these patients. Even though there are no environmental strains available from this ICU, the genetic similarity of all the clinical strains falling into cluster 1 is striking, with a minimal SNP distance of 1 SNP (among strains 10, 17, and 26) (Supplementary Table 4). This makes intra-hospital transmission a very likely scenario, suggesting that the outbreak strain may have resided in the hospital environment after discharge of patient 2, from where it was transmitted to patient 3. This is also supported by the finding that both patient 2 and 3 were culture negative for *P. aeruginosa* (including XDR-PA) in all samples taken on the day of admission (day 0), and only became positive for XDR-PA several days or weeks later (patient 2: on day 3, and patient 3: on day 20).

In cluster 2, all strains originated from hospital B. Interestingly, all the environmental strains from this hospital phylogenetically fall into cluster 2. This confirms that there is a reservoir in the hospital environment in regard to the outbreak strain from cluster 2.

Cluster 3 and cluster 4 correspond to one individual patient each, both having been admitted from abroad (patient 22 from Kuwait and patient 23 from Egypt, respectively). This indicates an introduction of these strains into our hospitals. Cluster 5 consists of only one single distinct isolate (patient 9), i.e., no other strains sampled from this patient were included in this study. Cluster 6 corresponds to one individual patient (hospital A). Cluster 7 corresponds to two different patients (hospital A and B). Cluster 8 represents three strains from one individual patient (hospital B). Interestingly, these three strains (strain ID 53, 54, and 55) are relatively closely related to cluster 1, in that they seem to cluster in the core genome phylogeny, whereas further analysis revealed that they differ significantly with a minimum SNP difference of 2,006 SNPs as compared to the remaining strains (Supplementary Table 4). The median SNP difference of these 3 strains was 2,057 SNPs, while the median SNP difference among the remaining strains was 14 SNPs. Thus, these three strains were considered to constitute an independent cluster outside cluster 1.

Overall, there were only two patients from whom not all strains phylogenetically grouped into one single cluster. For patient 2 (*n* = 9), most strains fall into cluster 1 (*n* = 6), but there are also strains falling into cluster 6 (*n* = 3). For patient 3 (*n* = 21), all but one strain fall into cluster 2 (*n* = 20), while one strain falls into cluster 7. This suggests that these patients harbored different *P. aeruginosa* strain lineages.

### Mutations Within the Core Genome Reveal SNPs in Mercury Detoxification Genes of Cluster 2

To elucidate the Intra-hospital molecular evolution of the *P. aeruginosa* outbreak strains on the single gene level, single nucleotide polymorphism (SNP) and insertion-deletion (indel) calling was performed. To this end, concomitant analysis of both environmental and clinical strains in parallel is crucial, with the assumption that the prolonged outbreak over 4 years had been maintained by regular transmission of strains between hospital environment and patients. Thus, the following analyses focus on cluster 2, containing both environmental and clinical strains.

A prevalence filter was applied to identify the most frequent SNPs and indels, and mutations were only reported if they occurred in at least 15% of strains due to their high prevalence and thus more significant relevance. In order to assess the impact of a SNP or indel on the protein function, we furthermore analyzed the functional effects of these mutations. Functional effect analysis is crucial in order to assess the actual impact of a SNP on the functionality of the translated protein, i.e., translating the change on the gene level to the protein level. Neutral functional impact of an amino acid modification generally implies that even though a gene is affected by genetic mutation, it is nevertheless evolutionarily conserved, in that the functionality of the coded protein is retained, e.g., because of a synonymous mutation leading to a nucleotide exchange but not resulting in a change of the coded amino acid. However, even non-synonymous mutations leading to a change in the coded amino acid may have a neutral functional impact, in cases where the amino acid change does not affect protein function. In contrast, deleterious functional impact of an amino acid modification implies that the coded protein is not functional anymore, e.g., because of a non-synonymous mutation leading to an amino acid exchange which affects functionality, depending on the position of the SNP and the structure of the respective protein.

Overall, cluster 2 features 19 SNPs after prevalence filtering (Table 1). Within these, there are SNPs with both neutral but also with deleterious functional impact (Table 1). Among the former, likely to be evolutionarily conserved for their function, there are an integrase (A0A220P6P1), a putative allantoin permease (A0A0P1D6C1) and an uncharacterized protein annotated as phosphorelay sensor kinase activity (GO:0000155). Among the latter, there are two genes involved in regulation of a multidrug efflux pump and in a mercury detoxification operon, namely MexZ (Q9ZH26) and MerR family transcription regulator (A0A1S1BX29). Apart from SNPs, there are only two genes affected by indels in cluster 2 after prevalence filtering, all of which predicted to have a neutral effect on protein function (Table 2).

**Table 1:** SNPs above the prevalence filter (>15%/<85%).

**UniProt BLAST annotation**		**General information**		**PROVEAN**	**GO term**		
**Protein name**	**UniProt acc. no**.	**Prevalence**	**Variant**	**Prediction**	**no**.	**Aspect**	**Info**
Phospho-N-acetylmuramoyl-pentapeptide- transferase	A0A220PD53	19.0%	V340G	Deleterious	–	–	–
Undecaprenyldiphospho- muramoylpentapeptide beta-N-acetylglucosaminyltransferase	A0A220PD36	20.7%	T148A	Neutral	–	–	–
Dienelactone hydrolase family protein	A0A1Y0GE31	19.0%	**–**	**–**	GO:0016787	F	hydrolase activity
Type VI secretion system Vgr family protein	A0A1G5LY91	19.0%	Q591L	Deleterious	–	–	–
Uncharacterized protein	A0A1C7C392	20.7%	–	–	–	–	–
**Putative allantoin permease**	A0A0P1D6C1	15.5%	F393L	Neutral	GO:0005215	F	Transporter activity
					GO:0055085	P	Transmembrane transport
Putative aldose 1-epimerase	A0A220PGB1	15.5%	Q268H	Deleterious	–	–	–
ATP synthase epsilon chain	Q9HT21	15.5%	G67A	Deleterious	GO:0046933	F	Proton-transporting ATP synthase activity, rotational mechanism
					GO:0015986	P	ATP synthesis coupled proton transport
Uncharacterized protein	A0A1G5M0Q6	15.5%	T106P	Neutral	–	–	–
**Integrase**	A0A220P6P1	20.7%	K219N	Neutral	–	–	–
Acyl-[acyl-carrier-protein]–UDP-N- acetylglucosamine O-acyltransferase	Q9X6P4	15.5%	Q100E	Deleterious	GO:0008780	F	Acyl-[acyl-carrier-protein]-UDP-N-acetyl- glucosamine O-acyltransferase activity
					GO:0009245	P	Lipid A biosynthetic process
					GO:0009103	P	Lipopolysaccharide biosynthetic process
Putative transcriptional regulator	A0A220PB10	17.2%	L269S	Neutral	–	–	–
Phosphotransferase family protein	A0A220PAS8	20.7%	Q221^*^	Neutral	–	–	–
Type VI secretion system Vgr family protein	A0A1G5KZC8	19.0%	M411R	Deleterious	–	–	–
**Uncharacterized protein**	A0A1D5BW36	20.7%	N165D	Neutral	GO:0000155	F	**Phosphorelay sensor kinase activity**
**MexZ**	Q9ZH26	19.0%	L123^*^	Deleterious	GO:0003677	F	DNA binding
					GO:0006355	P	Regulation of transcription, DNA-templated
					GO:0006351	P	Transcription, DNA-templated
**MerR family transcriptional regulator**	A0A1S1BX29	15.5%	A115T	Deleterious	GO:0003677	F	DNA binding
					GO:0006355	P	Regulation of transcription, DNA-templated
Uncharacterized protein	A0A220PHL6	17.2%	–	–	–	–	–
Uncharacterized protein	A0A220PHL6	17.2%	–	–	–	–	–

*Cluster 2: 19 SNPs*.

*Only SNPs above the prevalence filter (prevalence in at least 15% of strains) and with resulting change in coded amino acid are reported, i.e., SNPs not resulting in amino acid change (non-coding SNPs) are omitted from this table. Variant annotation was performed by SnpEff. Protein function prediction was performed by PROVEAN protein with a default cutoff of −2.5. If variant is not coding, information about “variant” (general information) and “prediction” (PROVEAN) is not indicated in the table*.

*UniProt BLAST annotation: the best hit (by identity) is given in the table*.

*Bold print: Proteins discussed in the manuscript text*.

* *(asterisk) = translation termination (stop) codon*.

*UniProt acc. no., UniProt accession number; no., number*.

**Table 2:** Indels and frameshifts with high or moderate impact and above the prevalence filter (>15%/<85%).

**UniProt BLAST annotation**		**General information**		**PROVEAN**	**GO term**			**SnpEff annotation**			
**Protein name**	**UniProt acc. no**.	**Prev**.	**% prot**.	**Prediction**	**No**.	**Asp**.	**Info**	**Variant**	**Cat**.	**Annotation (effect)**	**Put. imp**.
Uncharacterized protein	A0A1G5IYU0	48%	87%	Neutral	No GO	–	–	p.Leu252_Glu253insAla	I	Disruptive_inframe_insertion	M
Gamma-glutamylputrescine oxidoreductase	A0A1F0J8L6	20%	1%	Neutral	GO:0016491	F	Oxido-reductase activity	p.Thr4_Ser5insProTyrPro	I	Conservative_inframe_insertion	M
			1%	na				p.Ser5fs	FS	Frameshift_variant	H

*Cluster 2: 2 indels and frameshifts*.

*Only indels and frameshifts above the prevalence filter (prevalence in at least 15% of strains) are reported. Variant annotation was performed by SnpEff. Protein function prediction was performed by PROVEAN with a cutoff of −2.5 (default)*.

*“% of protein” (general information) = percent of total protein length before frameshift or termination; “variant category” (SnpEff): manually assigned based on HGVS.c/HGVS.p annotation; “HGVS.c & HGVS.p” = variant using HGVS (Human Genome Variation Society) notation with 1- or 3-letter amino acid code. Note that in case the variant is not coding, HGVS.p (protein level) is given instead of HGVS.c (DNA level)*.

*UniProt BLAST annotation: the best hit (by identity) is given in the table*.

*prev., prevalence; % prot., % of protein; asp., aspect; cat., category; put. imp., putative impact; na, not applicable; UniProt acc. no.; UniProt accession number; no., number; I, indel (category); FS, frameshift (category); M, moderate (putative impact); H, high (putative impact)*.

### Analysis of Cluster 2 Reveals High Genomic Plasticity in the Accessory Genome

In order to further dissect the molecular evolution history within cluster 2, the corresponding accessory genome was analyzed accordingly. As the accessory genome consists of all the genes which not all strains of a cluster have in common, the biggest and most relevant differences regarding molecular evolution lie in the accessory genes, representing genes which have either been acquired or lost by some strains in comparison to the other strains. This allows for molecular evolution analysis over time. Even though the strains within a cluster are phylogenetically closely related to each other, they harbor considerable numbers of accessory genes which were analyzed.

**Figure 3:**
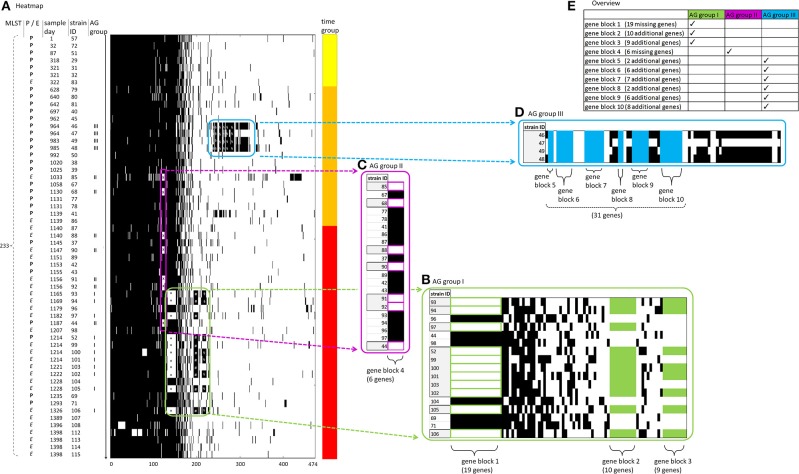
Accessory genome of cluster 2. All strains of cluster 2 (*n* = 59) are shown. Heatmap visualization with gene presence indicated by black bars and gene absence by white bars. Frames contain genes pertaining to AG group I, II, and III (in green, violet, and blue, respectively) and are presented in magnification inserts **(B–D)** next to the heatmap **(A)**. An overview of AG groups and gene blocks is given in the table insert **(D)**. **(A)** *X axis*: accessory genes are consecutively numbered, beginning with 1 through 474. *Left Y axis*: indication of (i) the source of the strains [P, patient (bold) or E, environment (italic)] (“P/E”), (ii) the MLST type (“MLST”), (iii) day of sampling (“sample day”) consecutively counted with the day of sampling of the oldest strain in this study (strain ID 57) set to 1, (iv) strain identification number (“strain ID”), chronologically ordered by timepoint of sampling descending from early to late, and (v) AG groups I, II and III (“AG group”). *Right Y axis*: assignment of the strains to three time groups corresponding to early, middle and late time periods of sampling (yellow, orange and red, respectively). **(B–D)** *Magnification inserts*: AG group I, II and III (in green, violet, and blue, respectively) with indication of gene blocks 1–0 and the number of genes contained within these. Missing gene blocks are framed in the respective color, whereas additional gene blocks are filled in the respective color. Genes not belonging to one of the gene blocks are filled in black color. For AG groups I and II, some additional strains (without genes belonging to one of the gene blocks) are displayed in the magnification inserts for comparative visualization and are filled in black color. Abbreviations: AG group, accessory genome group; MLST, multi-locus sequence typing.

In the following analysis of the accessory genome of cluster 2, patterns of gene presence or absence were explored and displayed in a heatmap (Figure 3). Genes gained or lost were subsumed to “gene blocks,” whereas the affected strains were subsumed to “accessory genome groups” (“AG groups”).

In total, cluster 2 harbors 474 accessory genes, indicating high genomic plasticity. As much as one third of these accessory genes are shared by the vast majority of strains. In order to provide further analysis of possible horizontal gene transfer (HGT), the G+C content of the genes subsumed to AG groups was calculated. Interestingly, the G+C content of these genes (mean = 59.36 %) differed in comparison to the G+C content of the whole genome sequences of the strains (mean = 65.71%), thus providing evidence about HGT (Supplementary Table 5).

AG group I consists of 11 strains and contains gene blocks 1–3 (Figure 3, table insert). One of these strains was patient-derived (strain ID 52, patient 5), whereas the others were of environmental origin. All 11 strains lack one block of 19 genes (gene block 1) and most harbor two additional blocks of 10 and 9 genes (gene block 2 and gene block 3), respectively. All these genes are presented in Supplementary Tables 5a–c, respectively.

In gene block 1, two genes were annotated as helicases [DNA helicase (A0A1G5KZ05), and ATP-dependent helicase HepA (A0A1G5L015)]. Other genes in gene block 1 such as an integrase (W8QZ61), a putative phage-type endonuclease (A0A1G5KYJ0) and a phage/plasmid-like protein (A0A1G5L075) indicate horizontal gene transfer. Further genes in this gene block include a heat shock protein 70 (A0A1G5KZ28) and a DNA repair protein RadC (A0A1G5KYU0).

In both gene block 2 and gene block 3, most genes are annotated as uncharacterized proteins, except for a membrane protein (W1MQ59). This protein is an integral component of membrane, according to the GO annotation (GO:0016021), and this annotation is shared by another three uncharacterized proteins in both gene block 2 and gene block 3 (W1MPI7, B3G281, and A0A0H3QL15). Nine genes in gene block 2 are duplicated in gene block 3 within eight strains. In order to exclude an assembly artifact we performed an analysis of the genomic environment of the respective genes. Their surrounding regions proved to be different between the corresponding genes from gene block 2 and gene block 3.

AG group II consists of seven strains and contains only one gene block (gene block 4). Out of these seven strains, five were of environmental origin and two patient-derived. Gene block 4 consists of six genes, among them a putative integrase (A0A220P3T3) (Supplementary Table 5d).

AG group III consists of four strains and contains gene blocks 5–10. All these four strains were sampled from the same patient, harboring a fairly large number of additional accessory genes (Supplementary Table 5c). There are two more strains (strain ID 45, 50) from this patient included in this study which, in contrast to the four strains in AG group III, do not possess any of these additional accessory genes. Among the additional genes in gene blocks 5–10, there is a resistance gene [AacA4 (A0A223LNV7)] as well as several genes related to horizontal gene transfer: conjugal transfer protein (A0A140SAI9), lytic transglycosylase (A0A140S8U1), integrating conjugative element protein (A0A1Q9R4V8), and prophage CP4-57 regulatory protein (AlpA) (A0A0N9ZY07).

In summary, there is an enormous dynamic of accessory genes within the strains of the monophyletic cluster 2. Comparing the strains over time, it is evident that large amounts of genetic material have both been acquired and lost during evolution, reflecting the adaptability of the *P. aeruginosa* genome. This unexpectedly high genetic variability indicates high genomic plasticity, likely mediated by horizontal gene transfer.

### Protein Enrichment Shows Virulence-Associated Proteins Enriched in the Late Time Group of Cluster 2 Strains

Aiming at analysis of genomic differences having occurred over time, the isolates within each cluster were assigned to time groups corresponding to early, middle, and late time periods of sampling during the course of the outbreak (see Methods section). Cluster 2 was divided into three time groups. These time groups were subsequently compared to each other. To this end, two complementary methods were applied, namely protein enrichment analysis and functional enrichment analysis.

**Figure 4:**
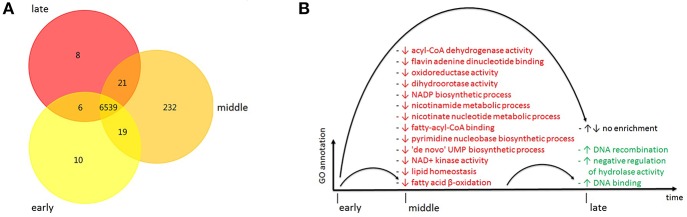
**(A)** Protein enrichment of cluster 2, Venn diagram visualization of proteins shared between time groups corresponding to early, middle and late time periods of sampling (yellow, orange and red, respectively) during the course of the outbreak. **(B)** Functional enrichment of GO terms within cluster 2, visualization of GO term names overrepresented or underrepresented in time groups corresponding to early, middle and late time periods of sampling during the course of the outbreak. Enrichment (overrepresentation): ↑ (green), depletion (underrepresentation): ↓ (red). GO, gene ontology.

In protein enrichment, protein annotation is obtained by BLASTX analysis for each of the time groups individually, and subsequently the presence or absence of individual proteins between different time groups is compared. Thus, protein enrichment analysis provides resolution on the level of single proteins. As evident from the Venn diagram visualization, there are 6,539 proteins shared by all the strains within cluster 2, while a smaller number of proteins is unique to individual time groups (Figure 4A). Cluster 2 has 10,232, and 8 unique proteins in its early, middle and late time group, respectively, amounting to a total of 251.

As the early and late time groups span the longest time frame in this study, they can be expected to best reflect long-term evolution and were thus further analyzed in detail (Supplementary Table 6). On the one hand, several proteins related to DNA processing and binding are only present in the early time group and thus can be assumed to have been lost over time [DNA polymerase III subunit gamma/tau (A0A0H2Z990), DNA polymerase III subunits gamma and tau (A0A157WPG4), and nucleic acid-binding protein (A0A1F0IY03)] (Supplementary Table 6a). On the other hand, transposases (A0A0U3JKU6, A0A0F7R4E2) are among the proteins only present in the late time group, just as a Hcp1 family type VI secretion system (T6SS) effector (A0A157WVX6) and a FHA domain-containing protein (A0A1S1CAI3) (Supplementary Table 6b). These genes can be assumed to have been gained over time. Overall, this strongly suggests the acquisition and loss of mobile genetic elements by the cluster 2 strains sampled over the course of the outbreak. In addition to these unique proteins in either the early or the late time group, there are also a few shared proteins present in both the early and the late time group. These genes comprise proteins such as a cold shock protein (A0A0V5G2H1) a transcriptional regulator (A0A1E9D6X8) and a TetR family transcriptional regulator (A0A1F0IIG9), but also proteins relevant for conjugal transfer and/or plasmids [Conjugal transfer protein (A0A1V6KPI1) and RAQPRD family plasmid (A0A1V6KP88)] (Supplementary Table 6c). The vast majority of these proteins were present in all strains constituting the early, late or combined early and late time group of cluster 2 (Supplementary Table 6, Supplementary Figure 1).

### Functional Enrichment Shows Significant Changes in Molecular Function and Biological Processes

Functional enrichment analysis was based on the same time group assignment of the strains as for protein enrichment analysis and also described in the Methods section. In functional enrichment, functional information is assigned to the individual genes by the means of gene ontology (GO) terms as defined by the Gene Ontology consortium, allowing the subsequent comparison of categories of gene function. The different time groups are compared to each other on the level of GO term frequency differences, resulting in lists of functional categories which are each calculated to be more abundant (overrepresentation) or less abundant (underrepresentation) between two time groups. This allows drawing conclusions about the respective functional properties having either been acquired or lost during evolution.

In contrast to protein enrichment analysis, functional enrichment cannot provide resolution of changes in individual proteins. However, the advantage of functional enrichment analysis lies in its ability to detect whether or not there is redundancy of protein functions in the strains over time. Thus, compensatory changes are taken into account and only significant gain or loss of functional metabolic processes is considered. This allows analysis of the big picture, only identifying those adaptive changes in function which are not compensated for by redundancy in an organism.

Most of the GO terms are underrepresented in the middle time group as compared to the early time group, while three GO terms are overrepresented in the late time group as compared to the middle time group (Figure 4B). These GO terms are associated with NAD+/acyl-CoA metabolism [acyl-CoA dehydrogenase activity (GO:0003995), fatty-acyl-CoA binding (GO:0000062), and fatty acid beta-oxidation using acyl-CoA dehydrogenase (GO:0033539)]. When looking at the distribution of the GO terms over the three GO aspects, there is an almost equal number pertaining to “molecular function” (*n* = 7) and “biological process” (*n* = 9) (Supplementary Table 7).

## Discussion

This study was initiated with the aim to elucidate an outbreak of bla_VIM_-carrying XDR *P. aeruginosa* involving two adjacent hospitals. We focused on comparative WGS analysis of all available strains over a time course of 4 years to (i) investigate genetic relatedness and to (ii) identify genetic evidence of evolution including adaptation to the hospital setting where the suspected monoclonal outbreak had been ongoing for several years.

Interestingly, subsequent WGS data analysis in this study revealed the core genome of all 100 strains to contain as many as 94,696 SNPs. As this would reflect a much higher rate of structural variation than expected from a monoclonal outbreak (Feliziani et al., 2014), further phylogenetic analysis was performed and revealed grouping into several clusters, with most strains falling into one of two major clusters (cluster 1 and cluster 2). All strains constituting cluster 1 were of clinical origin, while the strains constituting cluster 2 were of both clinical and environmental origin. Thus, only cluster 2 is further discussed in detail, with this study aiming at in-hospital molecular evolution analysis involving patients and the hospital environment as a reservoir alike, for which analysis of both clinical and environmental strains in parallel is required. Evolutionarily speaking, not only is adaptation to environmental conditions influencing the molecular evolution of the strains, but evolutionary intra-patient selection of virulence determinants is also likely to affect the molecular evolution. As a prerequisite for an ongoing outbreak, the strains have to acquire or at least keep their ability to be frequently transmitted to patients and establish colonization and/or infection.

Relevant core genome mutations in cluster 2 were deleterious in eight genes and neutral in seven genes (Table 1). Out of the eight deleterious SNPs, two SNPs are located in genes regulating bacterial resistance and stress response (*mexZ* and *merR*). The *mexZ* gene is affected by a deleterious SNP in a significant percentage of strains (19.0 %) in cluster 2. *MexZ* is a gene regulator controlling the expression of *mexXY*, components of a multidrug efflux pump responsible for *P. aeruginosa* resistance to several antibiotics (Alguel et al., 2010). As MexZ acts as a repressor to block transcription initiation of MexXY, a functionally deleterious SNP in the *mexZ* gene likely results in constitutive expression of *mexXY*. Thus, this mutation both contributes to an antibiotic resistance phenotype and also constitutes a survival advantage for the strains affected, as evident from data on clinical cystic fibrosis (CF) *P. aeruginosa* isolates (Smith et al., 2006; Jahandideh, 2013). Interestingly, the *mexZ* SNP is distributed over the whole course of the outbreak being present in strains of the early, middle and late time periods of sampling [1, 3, and 7 strain(s), respectively].

Another deleterious SNP is present in the *merR* family transcription regulator which tightly controls expression of the *mer* operon, conferring bacterial resistance to inorganic mercury and organomercurials (sensing, transport, and detoxification of these cytotoxic agents) (Chang et al., 2015). As MerR acts as a repressor to block transcription initiation of the *mer* operon, a functionally deleterious SNP in the *merR* gene likely results in constitutive expression of the *mer* operon. We assume that this would increase the respective strains' ability to withstand environmental stressors, possibly related to other cytotoxic agents beyond mercury, as is known for a negative transcription regulator in the *merR* family for *Neisseria gonorrhoeae* and *Haemophilus influenzae* (Supa-Amornkul et al., 2016). In this light, this mutation can be seen as a gain-of-fitness determinant especially in the hospital environment with exposure to various disinfectants and cleaning agents. With the exception of one strain sampled from a patient, all other strains affected by the SNP in the *merR* gene were sampled from the environment, and all strains fall into the late time group. Thus, the MerR mutation seems not only enriched in environmental strains over clinical strains, but was also only found in strains during the late time period of the outbreak, indicating an adaptation to environmental stress, potentially even disinfectants.

Out of the seven neutral SNPs in the core genome of cluster 2, two SNPs are discussed here as they are likely to be important for genetic mobility or virulence, thus explaining that only functionally neutral mutations have occurred in these genes. The first affected gene is an integrase, which mediates unidirectional site-specific recombination between two DNA recognition sequences. Integrases indicate infection of a bacterial strain by a phage, either as a past or recent event, and it is likely for other genes to become transduced along with integrases (Groth and Calos, 2004). The second gene is a putative allantoin permease annotated to exert transporter activity and transmembrane transport and likely of importance for the uptake of substrates or for export of toxic agents. Allantoin is a major metabolic intermediate in the degradation of purine nucleobases.

In addition to these core genome SNPs, differences in the accessory genomes of the clusters were investigated in this study.

In cluster 2, there are three accessory genome groups (AG groups) of strains which are characterized by gain and loss of unique gene blocks (each comprising 6–31 genes). Overall, these gene blocks contain many genes which are indicative of horizontal gene transfer, and a selection of these genes is discussed following on. In gene block 1, we found two helicases to be missing in comparison to all other strains of cluster 2. Helicases are enzymes unwinding DNA, classified in 6 groups (superfamilies), and are not only involved in nucleic acid replication but also in DNA/RNA turnover and repair as well as in stress resistance in general (Singleton et al., 2007; Jarmoskaite and Russell, 2014). ATP-dependent helicase HepA belongs to superfamily 2, and is known to be necessary for DNA repair from UV light and possibly other sources such as free radicals and alkylating agents (Muzzin et al., 1998; Coleman et al., 2000). Thus, the lack of helicases in these strains may reflect an indirect gain of fitness, as hypermutability may lead to the occurrence of novel successful mutant strains, while it has also been shown that helicases may confer a higher risk of a fatal outcome in patients with *P. aeruginosa* sepsis (Willmann et al., 2018). Another protein which has gone missing from the accessory genomes of the 11 strains in AG group I is heat shock protein 70. Heat shock proteins (Hsps) not only play a role in the protein folding machinery of cells, but also in stress protection (heat stress as well as toxic chemicals). Furthermore, DNA repair protein RadC is missing in AG group I. This protein had been implicated in repair of DNA strand breaks, however this implication has been questioned, which explains why there is currently no GO annotation available, thus making it difficult to interpret its functional relevance (Felzenszwalb et al., 1986; Attaiech et al., 2008).

In general, DNA binding and other DNA-related enzymatic functions are among the GO annotations of all the proteins in AG group I, in line with the DNA repair and stress response functions of the above mentioned individual proteins. Likewise, several of the GO term annotations of most proteins in AG group I relate to ATP binding, indicating that the missing proteins are involved in metabolic processes consuming ATP and are likely to belong together functionally. The loss of DNA repair genes usually results in a faster accumulation of mutations. While many of these spontaneously occurring mutations are likely detrimental for the fitness or survival, some are likely to exert beneficial genomic changes. Thus, strains with higher mutation rates may have a better chance to keep adapting to the environmental stress they are exposed to in the hospital environment.

Among the proteins missing in gene block 1, there are also three genes related to phage integration, namely a putative phage-type endonuclease, a phage/plasmid-like protein and an integrase. In this light, it is very likely that a prophage was excised from the genome of the strains pertaining to gene block 1, taking along with it several genes, while it remained integrated (lytic state) into the bacterial genomes of the other strains of cluster 2. Phage infection may even confer advantage to the respective strain, as it has been shown in *P. aeruginosa* that phages often provide their host with defense against other types of phages by manipulating the respective host receptors (Ofir and Sorek, 2018).

There are also phage-related genes in AG group II (gene block 4) and AG group III (gene blocks 5–10). For example, in AG group II, a putative integrase gene is missing in comparison to all other strains of cluster 2, making it likely that this gene has been excised by a phage having taken along some other genes with it. In AG group III (gene blocks 5–10), a resistance gene (*aacA4*) has been acquired which is not present in all other strains of cluster 2. In addition to this gene, several other genes related to phage integration have been acquired (conjugal transfer protein, lytic transglycosylase, integrating conjugative element protein, and prophage CP4-57 regulatory protein (AlpA). Therefore, it is likely that the *aacA4* resistance gene could have been transferred along with the other genes by horizontal gene transfer. In summary, horizontal gene transfer can be assumed as the main driving force behind the genetic plasticity outlined above, as supported by the differing G+C content of the respective genes in comparison to the G+C content of the whole genome sequences of the strains.

In protein enrichment analysis of cluster 2, relatively few proteins were unique to the strains of either the early time group (10 proteins) or the late time group (8 proteins). Among them, Hcp1 family type VI secretion system effector was identified to be unique to the late time group. This protein constitutes one of the components of the type 6 secretion system (T6SS) macromolecular machines (Ruiz et al., 2015). Also a FHA domain-containing protein is only present in the late time group, and both eukaryotes and prokaryotes possess various proteins with one or more such FHA domains. For example, *Mycobacterium tuberculosis* has been shown to possess an ABC transporter protein containing two FHA domains (Curry et al., 2005; Spivey et al., 2011). Thus, it is not unlikely that FHA domain-containing proteins might be part of ABC transporters in other bacteria and confer antibiotic resistance. There is limited data on *P. aeruginosa*, with one study suggesting that FHA is a core scaffolding protein of the pseudomonal T6SS (Mougous et al., 2007).

However, it is not only these unique proteins in the late time group that can provide insights into molecular evolution, but also those proteins which are shared between time groups. The early and late time groups of strains share six identical proteins with each other, including a cold shock protein. Cold shock proteins (Csps) are typically induced in response to cold environmental temperatures, while some Csps are non-cold inducible and rather involved in various cellular processes to promote stress adaptation responses (Horn et al., 2007; Keto-Timonen et al., 2016). In addition, a probable cold shock protein has previously been identified in *P. aeruginosa* amongst other genomic segments to be associated with aminoglycoside resistance (Struble and Gill, 2009). Thus, Csps in the outbreak strains likely provide not only protection against stressors, but could also contribute to maintaining antibiotic resistance. Another protein shared by both the early and the late time group of strains is the TetR family transcriptional regulator. TetR is a repressor of TetA: an efflux pump in the membrane removing tetracycline and other toxic substances from the bacterial cell. In the presence of tetracycline, the target operator repression of TetR is released, resulting in tetracycline resistance by activation of the respective efflux pump (Ramos et al., 2005).

In order to assess functional plasticity of the outbreak strains over time, functional enrichment was investigated. Functional enrichment analysis of cluster 2 identified various GO terms, most of which are overrepresented in the early time group. Interestingly, several of these GO terms were associated with acyl-CoA metabolism. This metabolism is part of the fatty acid degradation pathway of *P. aeruginosa* which in turn is involved in nutrient acquisition in biofilm growth (Zarzycki-Siek et al., 2013). It can be hypothesized that these metabolic activities have proven to be dispensable for the survival within the hospital and thus are underrepresented in the middle time group in comparison to the early time group, even though there is no difference in functional enrichment between the early and the late time group.

In conclusion, whole-genome sequencing technology enabled us to reveal a polyclonal outbreak of XDR *P. aeruginosa* with two major clusters of strains. Furthermore, we detected remarkable genomic dynamics and adaptation of *P. aeruginosa* strains over a 4-year period within two hospitals, most likely primarily driven by mobile genetic elements. Genomic alterations affected bacterial stress response, virulence, transport function and mercury detoxification, and thus present possible explanations for the successful persistence and spread of these strains. Our study is a step forward toward a better understanding of the molecular evolution of *P. aeruginosa* in a “real-world” hospital setting, taking us closer to personalized medicine with our efforts to identify what genetic determinants actually make outbreak strains so successful, and allowing us to learn from the genetic adaptation of these strains. The knowledge of such evolutionary trajectories is key to more specific and tailored infection control measures and will help us fight nosocomial pathogens and their global spread. These data adjust our understanding of outbreak strain persistence and virulence in the context of molecular medicine, which could eventually translate into future strategies and allow for the development of new approaches for infection prevention and control measures, and increase targeted action to tackle XDR *P. aeruginosa* outbreaks. Further studies that investigate the genomic resilience of *P. aeruginosa* are warranted.

## Materials and Methods

### Hospital Setting, Sample Collection, and Infection Control Measures

Active screening cultures for multidrug-resistant Gram-negatives including *Pseudomonas aeruginosa* were performed at both hospitals during the study period by weekly rectal and pharyngeal swabs and stool samples. Cetrimide agar (Becton, Dickinson and Company, Le Pont-de-Claix, France) was used for selective cultivation of *P. aeruginosa*.

Strains were included in this study if they had an XDR phenotype and harbored the bla_VIM_ gene, amounting to 100 strains over a period of 4 years. All strains were recovered from two adjacent hospitals in Germany [a 330-bed casualty hospital (hospital A), and a 1500-bed teaching hospital (hospital B)]. The strains were routinely sampled from patients (colonization and infection) in hospital A and B, as well as from the hospital environment (mostly toilets and washing basins) in hospital B. The majority of strains originated from either the ICU in hospital A specialized in burn patients and other casualties, or from the hemato-oncology wards from hospital B with many patients undergoing hematopoietic stem cell transplantation (HSCT).

During the prolonged outbreak going on over 4 years, infection prevention and control (IPC) measures were intensified including technical measures. In the hemato-oncology wards, these measures included weekly active screening cultures for *P. aeruginosa* with immediate isolation of case patients and isolation of contact patients until three negative screening results.

Beyond this, also technical measures were undertaken, namely equipment of washing basin plugholes with cap outlets to prevent splashing water and aerosol formation, and all siphons under the washing basins had been replaced and were equipped with thermal disinfection devices (93°C, with additional vibration based cleaning at 50 Hz; Biorec/Moveomed, Lauta/Radebeul, Germany). Moreover, professional environmental cleaning with blue light was initiated twice yearly (Maclean et al., 2010).

In addition to weekly disinfection of all sanitary regions and weekly application of tube cleaners, comprehensive room disinfection after discharge of a case patient was also performed. For the latter, and particularly for the sanitary regions, a peracetic acid-based cleaning agent (Incidin Active, Ecolab Healthcare, Monheim, Germany) was used as of day 224 of the outbreak, instead of a glucoprotamin-based cleaning agent (Incidin Plus, Ecolab Healthcare) which had been used before. From day 278 of the outbreak onwards, a new cleaner (Into WC, Ecolab Healthcare) was applied to the siphons of all washing basins, showers and toilets once weekly.

### Microbiology and Origin of Strains

During the outbreak, all *Pseudomonas aeruginosa* isolates were cryopreserved at −80°C, regardless of their site of sampling (colonization and infection) or antibiotic susceptibility pattern. Identification of strains was performed by a linear MALDI-TOF mass spectrometer (AXIMA Assurance, bioMérieux, Marcy-l'Étoile, France and Biotyper, Bruker, Bremen, Germany), supplemented by Vitek 2 system identification (bioMérieux). Phenotypic resistance was determined by broth microdilution antimicrobial susceptibility testing on a Vitek 2 (bioMérieux), supplemented by disc diffusion and E-Test (Liofilchem, Roseto degli Abruzzi, Italy) according to EUCAST standards, available in Supplementary Table 9a^1^. Presence of bla_VIM_ genes was confirmed by PCR as described previously (Pitout et al., 2005).

### Whole Genome Sequencing

Genomic DNA extraction from pure cultures of 108 strains was performed by the Genomic-tip 100/G kit (Qiagen, Hilden, Germany) according to the manufacturer's Genomic DNA Handbook, and DNA concentration was determined fluorimetrically using a Qubit 2.0 fluorometer (Thermo Fisher, Dreieich, Germany). Whole genome sequencing (WGS) was performed on an Illumina HiSeq 2500 machine (rapid mode) with a 250 bp read length paired end approach and with a targeted 550 bp insert size (CeMeT GmbH, Tuebingen, Germany).

### Sequence Data Analysis

Sequence data was trimmed with Trimmomatic to a minimal length of 70 base pairs and otherwise default settings (Bolger et al., 2014). Genome assembly was performed by SPAdes (version 3.7.0) with “‘careful’ option” (Bankevich et al., 2012; Rozov et al., 2016). From the 108 WGS assemblies, eight were rigorously excluded because of data quality, leaving 100 strains with high assembly quality, as confirmed by contamination check and genome completeness calculation using CheckM version 1.0.13 (https://github.com/Ecogenomics/CheckM/wiki), available in Supplementary Table 8. The multi-locus sequence types of the strains were determined using the mlst software version 2.16.1 (https://github.com/tseemann/mlst). Genotypic resistance determinants were calculated by ResFinder (https://cge.cbs.dtu.dk/services/ResFinder/), available in Supplementary Table 9b. The core genome was calculated by Spine (version 0.1.2) with a 90% cutoff for concordance between homologous regions (Ozer et al., 2014). Phylogenetic analysis, in consideration of recombinational events, was calculated by Gubbins (version 2.1.0) with a maximum of 10 iterations (Croucher et al., 2015). Accessory genomes of the two major clusters identified in the phylogeny were calculated by Roary (version 1.006924) with 95% Blastp setting (Page et al., 2015). Genome annotation was performed by Prokka (version 1.11), and additional manual annotation was done using UniProt BLAST (Seemann, 2014; UniProt, 2017). In UniProt BLAST analysis, the best hit by identity was considered and is given in all the respective tables. For analysis of molecular evolution over time (functional and protein enrichment), the strains of cluster 2 were assigned to time groups representing early (day 1–322), middle (day 628–1,139), and late (day 1,140–1,398) time periods of sampling.

Functional enrichment was analyzed by a pipeline including CD-HIT EST (version 4.6) with a 90% cutoff for concordance and Blastn or Blastx with subsequent Blast2GO query or FunRich (version 4.1 and version 3.1.3, respectively) Venn analysis (Li and Godzik, 2006; Pathan et al., 2015). Calling of single nucleotide polymorphisms (SNPs) and insertion-deletion mutations (indels) was performed by SAMtools (version 0.1.19) and GATK tools (version 3.2-2) with a minimal mapping score of 30, and variant annotation by SnpEff (version 4.3), with subsequent functional effect analysis by PROVEAN (http://provean.jcvi.org/seq_submit.php) with a cutoff of −2.5 (Li et al., 2009; Mckenna et al., 2010; Choi et al., 2012; Cingolani et al., 2012).

## Ethics Statement

The study was conducted in accordance with the local ethics committee (No. 623/2018BO2, Ethik-Kommission an der Medizinischen Fakultät der Eberhard-Karls-Universität und am Universitätsklinikum Tübingen). The samples were obtained during routine care. No consent to participate was required, as no patient samples were analyzed, but bacterial strains only.

## Author Contributions

MW and IA designed the initial study setup. MB, CK, and AG performed wet lab work for NGS sequencing. MB and MW performed NGS data analysis. MB, SP, and MW gathered epidemiological data. MB wrote the initial manuscript, and MB and MW compiled the final manuscript, to which SP and IA contributed.

### Conflict of Interest Statement

The authors declare that the research was conducted in the absence of any commercial or financial relationships that could be construed as a potential conflict of interest.

## Footnotes

^1^The European Committee on Antimicrobial Susceptibility Testing. Breakpoint Tables for Interpretation of MICs and Zone Diameters, V (2018). Available online at: http://www.eucast.org/ast_of_bacteria/previous_versions_of_documents/.

## Abbreviations

AG group: accessory genome group
bla_VIM_: betalactamase VIM
CF: cystic fibrosis
DNA: desoxyribonucleic acid
ENA: European Nucleotide Archive
EUCAST: European committee on antimicrobial susceptibility testing
GO: gene ontology
HGT: horizontal gene transfer
HQ: high quality
ICU: intensive care unit
IPC: infection prevention and control (measures)
MALDI-TOF: Matrix-assisted laser desorption ionization-time of flight
MDR: multi-drug-resistant
MLST: multi-locus sequence typing
PFGE: pulsed-field gel electrophoresis
*P*.: *Pseudomonas*
PA: *Pseudomonas aeruginosa*
SNPs: single nucleotide polymorphisms
VIM: Verona integron-mediated metallobetalactamase (carbapenemase)
WGS: whole genome sequence/sequencing
XDR: extensive(ly) drug resistant/-ce
GEN: gentamicin
TOB: tobramycin
PIP: piperacillin
PIT: piperacillin-tazobactam
CTZ: ceftazidime
LEV: levofloxacin
CIP: ciprofloxacin
MER: meropenem
CEP: cefepime
FOS: fosfomycin
AZT: aztreonam
COL: colistin
AMI: amikacin.

## References

[B1] Alguel Y. Lu D. Quade N. Sauter S. Zhang X. (2010). Crystal structure of MexZ, a key repressor responsible for antibiotic resistance in *Pseudomonas aeruginosa*. J. Struct. Biol. 172, 305–310. 10.1016/j.jsb.2010.07.012 20691272

[B2] Attaiech L. Granadel C. Claverys J. P. Martin B. (2008). RadC, a misleading name? J. Bacteriol. 190, 5729–5732. 10.1128/JB.00425-08 18556794 PMC2519389

[B3] Azzopardi E. A. Azzopardi E. Camilleri L. Villapalos J. Boyce D. E. Dziewulski P. (2014). Gram negative wound infection in hospitalised adult burn patients–systematic review and metanalysis. PLoS ONE 9:e95042 10.1371/journal.pone.0095042 24751699 PMC3994014

[B4] Bankevich A. Nurk S. Antipov D. Gurevich A. A. Dvorkin M. Kulikov. (2012). SPAdes: a new genome assembly algorithm and its applications to single-cell sequencing. J. Comput. Biol. 19, 455–477. 10.1089/cmb.2012.0021 22506599 PMC3342519

[B5] Bolger A. M. Lohse M. Usadel B. (2014). Trimmomatic: a flexible trimmer for Illumina sequence data. Bioinformatics 30, 2114–2120. 10.1093/bioinformatics/btu170 24695404 PMC4103590

[B6] Buhl M. Peter S. Willmann M. (2015). Prevalence and risk factors associated with colonization and infection of extensively drug-resistant *Pseudomonas aeruginosa*: a systematic review. Expert. Rev. Anti. Infect. Ther. 13, 1159–1170. 10.1586/14787210.2015.1064310 26153817

[B7] Chang C. C. Lin L. Y. Zou X. W. Huang C. C. Chan N. L. (2015). Structural basis of the mercury(II)-mediated conformational switching of the dual-function transcriptional regulator MerR. Nucleic Acids Res. 43, 7612–7623. 10.1093/nar/gkv681 26150423 PMC4551924

[B8] Choi Y. Sims G. E. Murphy S. Miller J. R. Chan A. P. (2012). Predicting the functional effect of amino acid substitutions and indels. PLoS ONE 7:e46688. 10.1371/journal.pone.0046688 23056405 PMC3466303

[B9] Cingolani P. Platts A. Wang Le L. Coon M. Nguyen T. Wang L.. (2012). A program for annotating and predicting the effects of single nucleotide polymorphisms, SnpEff: SNPs in the genome of Drosophila melanogaster strain w1118; iso-2; iso-3. Fly 6, 80–92. 10.4161/fly.19695 22728672 PMC3679285

[B10] Coleman M. A. Eisen J. A. Mohrenweiser H. W. (2000). Cloning and characterization of HARP/SMARCAL1: a prokaryotic HepA-related SNF2 helicase protein from human and mouse. Genomics 65, 274–282. 10.1006/geno.2000.6174 10857751

[B11] Croucher N. J. Page A. J. Connor T. R. Delaney A. J. Keane J. A. Bentley S. D.. (2015). Rapid phylogenetic analysis of large samples of recombinant bacterial whole genome sequences using Gubbins. Nucleic Acids Res. 43:e15. 10.1093/nar/gku1196 25414349 PMC4330336

[B12] Curry J. M. Whalan R. Hunt D. M. Gohil K. Strom M. Rickman L.. (2005). An ABC transporter containing a forkhead-associated domain interacts with a serine-threonine protein kinase and is required for growth of *Mycobacterium tuberculosis* in mice. Infect. Immun. 73, 4471–4477. 10.1128/IAI.73.8.4471-4477.2005 16040957 PMC1201257

[B13] De Abreu P. M. Farias P. G. Paiva G. S. Almeida A. M. Morais P. V. (2014). Persistence of microbial communities including *Pseudomonas aeruginosa* in a hospital environment: a potential health hazard. BMC Microbiol. 14:118. 10.1186/1471-2180-14-118 24885173 PMC4049484

[B14] Feliziani S. Marvig R. L. Lujan A. M. Moyano A. J. Di Rienzo J. A. Krogh Johansen H.. (2014). Coexistence and within-host evolution of diversified lineages of hypermutable *Pseudomonas aeruginosa* in long-term cystic fibrosis infections. PLoS Genet. 10:e1004651. 10.1371/journal.pgen.1004651 25330091 PMC4199492

[B15] Felzenszwalb I. Sargentini N. J. Smith K. C. (1986). *Escherichia coli* radC is deficient in the recA-dependent repair of X-ray-induced DNA strand breaks. Radiat. Res. 106, 166–170. 10.2307/3576790 3517933

[B16] Groth A. C. Calos M. P. (2004). Phage integrases: biology and applications. J. Mol. Biol. 335, 667–678. 10.1016/j.jmb.2003.09.082 14687564

[B17] Horn G. Hofweber R. Kremer W. Kalbitzer H. R. (2007). Structure and function of bacterial cold shock proteins. Cell Mol. Life Sci. 64, 1457–1470. 10.1007/s00018-007-6388-4 17437059 PMC11138454

[B18] Jahandideh S. (2013). Diversity in structural consequences of MexZ mutations in *Pseudomonas aeruginosa*. Chem. Biol. Drug Des. 81, 600–606. 10.1111/cbdd.12104 23611117

[B19] Jarmoskaite I. Russell R. (2014). RNA helicase proteins as chaperones and remodelers. Annu. Rev. Biochem. 83, 697–725. 10.1146/annurev-biochem-060713-035546 24635478 PMC4143424

[B20] Juhas M. (2015). *Pseudomonas aeruginosa* essentials: an update on investigation of essential genes. Microbiology 161, 2053–2060. 10.1099/mic.0.000161 26311069

[B21] Keto-Timonen R. Hietala N. Palonen E. Hakakorpi A. Lindstrom M. Korkeala H. (2016). Cold shock proteins: A minireview with special emphasis on csp-family of enteropathogenic yersinia. Front. Microbiol. 7:1151. 10.3389/fmicb.2016.01151 27499753 PMC4956666

[B22] Li H. Handsaker B. Wysoker A. Fennell T. Ruan J. Homer N.. (2009). The sequence alignment/map format and SAMtools. Bioinformatics 25, 2078–2079. 10.1093/bioinformatics/btp352 19505943 PMC2723002

[B23] Li W. Godzik A. (2006). Cd-hit: a fast program for clustering and comparing large sets of protein or nucleotide sequences. Bioinformatics 22, 1658–1659. 10.1093/bioinformatics/btl158 16731699

[B24] Maclean M. Macgregor S. J. Anderson J. G. Woolsey G. A. Coia J. E. Hamilton K.. (2010). Environmental decontamination of a hospital isolation room using high-intensity narrow-spectrum light. J. Hosp. Infect. 76, 247–251. 10.1016/j.jhin.2010.07.010 20864210

[B25] Marvig R. L. Sommer L. M. Molin S. Johansen H. K. (2015). Convergent evolution and adaptation of *Pseudomonas aeruginosa* within patients with cystic fibrosis. Nat. Genet. 47, 57–64. 10.1038/ng.3148 25401299

[B26] Mckenna A. Hanna M. Banks E. Sivachenko A. Cibulskis K. Kernytsky A.. (2010). The genome analysis toolkit: a mapreduce framework for analyzing next-generation DNA sequencing data. Genome Res. 20, 1297–1303. 10.1101/gr.107524.110 20644199 PMC2928508

[B27] Mougous J. D. Gifford C. A. Ramsdell T. L. Mekalanos J. J. (2007). Threonine phosphorylation post-translationally regulates protein secretion in *Pseudomonas aeruginosa*. Nat. Cell Biol. 9, 797–803. 10.1038/ncb1605 17558395

[B28] Muzzin O. Campbell E. A. Xia L. Severinova E. Darst S. A. Severinov K. (1998). Disruption of *Escherichia coli* hepA, an RNA polymerase-associated protein, causes UV sensitivity. J. Biol. Chem. 273, 15157–15161. 10.1074/jbc.273.24.15157 9614128

[B29] Ofir G. Sorek R. (2018). Contemporary phage biology: from classic models to new insights. Cell 172, 1260–1270. 10.1016/j.cell.2017.10.045 29522746

[B30] Ozer E. A. Allen J. P. Hauser A. R. (2014). Characterization of the core and accessory genomes of *Pseudomonas aeruginosa* using bioinformatic tools spine and AGEnt. BMC Genom. 15:737. 10.1186/1471-2164-15-737 25168460 PMC4155085

[B31] Page A. J. Cummins C. A. Hunt M. Wong V. K. Reuter S. Holden M. T.. (2015). Roary: rapid large-scale prokaryote pan genome analysis. Bioinformatics 31, 3691–3693. 10.1093/bioinformatics/btv421 26198102 PMC4817141

[B32] Pathan M. Keerthikumar S. Ang C. S. Gangoda L. Quek C. Y. Williamson N. A.. (2015). FunRich: an open access standalone functional enrichment and interaction network analysis tool. Proteomics 15, 2597–2601. 10.1002/pmic.201400515 25921073

[B33] Pidot S. J. Gao W. Buultjens A. H. Monk I. R. Guerillot R. Carter G. P.. (2018). Increasing tolerance of hospital *Enterococcus faecium* to handwash alcohols. Sci. Transl. Med. 10:eaar6115. 10.1126/scitranslmed.aar6115 30068573

[B34] Pitout J. D. Gregson D. B. Poirel L. Mcclure J. A. Le P. Church D. L. (2005). Detection of *Pseudomonas aeruginosa* producing metallo-beta-lactamases in a large centralized laboratory. J. Clin. Microbiol. 43, 3129–3135. 10.1128/JCM.43.7.3129-3135.2005 16000424 PMC1169086

[B35] Ramos J. L. Martinez-Bueno M. Molina-Henares A. J. Teran W. Watanabe K. Zhang X.. (2005). The TetR family of transcriptional repressors. Microbiol. Mol. Biol. Rev. 69, 326–356. 10.1128/MMBR.69.2.326-356.2005 15944459 PMC1197418

[B36] Rozov R. Brown Kav A. Bogumil D. Shterzer N. Halperin E. Mizrahi I.. (2016). Recycler: an algorithm for detecting plasmids from *de novo* assembly graphs. Bioinformatics 33, 475–482. 10.1093/bioinformatics/btw651 28003256 PMC5408804

[B37] Ruiz F. M. Santillana E. Spinola-Amilibia M. Torreira E. Culebras E. Romero A. (2015). Crystal structure of Hcp from *Acinetobacter baumannii*: a component of the type VI secretion system. PLoS ONE 10:e0129691 10.1371/journal.pone.0129691 26079269 PMC4469607

[B38] Seemann T. (2014). Prokka: rapid prokaryotic genome annotation. Bioinformatics 30, 2068–2069. 10.1093/bioinformatics/btu153 24642063

[B39] Singleton M. R. Dillingham M. S. Wigley D. B. (2007). Structure and mechanism of helicases and nucleic acid translocases. Annu. Rev. Biochem. 76, 23–50. 10.1146/annurev.biochem.76.052305.115300 17506634

[B40] Smith E. E. Buckley D. G. Wu Z. Saenphimmachak C. Hoffman L. R. D'argenio D. A.. (2006). Genetic adaptation by *Pseudomonas aeruginosa* to the airways of cystic fibrosis patients. Proc. Natl. Acad. Sci. U.S.A. 103, 8487–8492. 10.1073/pnas.0602138103 16687478 PMC1482519

[B41] Spivey V. L. Molle V. Whalan R. H. Rodgers A. Leiba J. Stach L.. (2011). Forkhead-associated (FHA) domain containing ABC transporter Rv1747 is positively regulated by Ser/Thr phosphorylation in *Mycobacterium tuberculosis*. J. Biol. Chem. 286, 26198–26209. 10.1074/jbc.M111.246132 21622570 PMC3138270

[B42] Stefani S. Campana S. Cariani L. Carnovale V. Colombo C. Lleo M. M.. (2017). Relevance of multidrug-resistant *Pseudomonas aeruginosa* infections in cystic fibrosis. Int. J. Med. Microbiol. 307, 353–362. 10.1016/j.ijmm.2017.07.004 28754426

[B43] Struble J. M. Gill R. T. (2009). Genome-scale identification method applied to find cryptic aminoglycoside resistance genes in *Pseudomonas aeruginosa*. PLoS ONE 4:e6576. 10.1371/journal.pone.0006576 19907650 PMC2771283

[B44] Supa-Amornkul S. Chantratita W. Srichunrusami C. Janchompoo P. Chaturongakul S. (2016). Listeria monocytogenes MerR-like regulator NmlRlm: its transcriptome and role in stress response. Foodborne Pathog. Dis. 13, 369–378. 10.1089/fpd.2015.2101 27058117

[B45] Tatarelli P. Mikulska M. (2016). Multidrug-resistant bacteria in hematology patients: emerging threats. Fut. Microbiol. 11, 767–780. 10.2217/fmb-2015-0014 27196948

[B46] UniProt (2017). UniProt: the universal protein knowledgebase. Nucleic Acids Res. 45, D158–D169. 10.1093/nar/gkw1099 27899622 PMC5210571

[B47] Willmann M. Goettig S. Bezdan D. Macek B. Velic A. Marschal M. (2018). Multi-omics approach identifies novel pathogen-derived prognostic biomarkers in patients with *Pseudomonas aeruginosa* bloodstream infection. bioRxiv. 10.1101/309898

[B48] Zarzycki-Siek J. Norris M. H. Kang Y. Sun Z. Bluhm A. P. Mcmillan I. A.. (2013). Elucidating the *Pseudomonas aeruginosa* fatty acid degradation pathway: identification of additional fatty acyl-CoA synthetase homologues. PLoS ONE 8:e64554. 10.1371/journal.pone.0064554 23737986 PMC3667196

