# Supplementary material for: Molecular Evolution of Extensively Drug-Resistant (XDR) Pseudomonas aeruginosa Strains From Patients and Hospital Environment in a Prolonged Outbreak
Source: Front Microbiol. 2019 Aug 8;10:1742. doi: 10.3389/fmicb.2019.01742 (PMC6694792; doi:10.3389/fmicb.2019.01742)
Supplement: Supplementary file 1 [file Table_1.docx]

**Supplementary Table 1** Number of strains per patient included in this study and cluster(s) to which the strain(s) are phylogenetically allocated. The mean number of strains per patient was calculated based on the numbers presented.

| **patient ID** | **number of strains** | **cluster** |
| --- | --- | --- |
| **1** | **1** | **1** |
| **2** | **9** | **1 (6), 6 (3)** |
| **3** | **21** | **1 (20), 7 (1)** |
| **4** | **3** | **8** |
| **5** | **1** | **2** |
| **6** | **3** | **2** |
| **7** | **1** | **2** |
| **8** | **1** | **7** |
| **9** | **1** | **5** |
| **10** | **4** | **2** |
| **11** | **1** | **2** |
| **12** | **2** | **2** |
| **13** | **1** | **2** |
| **14** | **3** | **2** |
| **15** | **6** | **2** |
| **16** | **1** | **2** |
| **17** | **1** | **2** |
| **18** | **1** | **2** |
| **19** | **1** | **2** |
| **20** | **2** | **2** |
| **21** | **3** | **2** |
| **22** | **3** | **3** |
| **23** | **2** | **4** |
|  |  |  |
| **mean (clusters 1-8)** | **3.1** |  |
| **mean (cluster 1)** | **9.0** |  |
| **mean (cluster 2)** | **2.1** |  |

**Supplementary Table 2** Gubbins exclusions.

Isolates excluded from the Gubbins phylogeny because of genetical identity to another isolate included in the Gubbins phylogeny, with additional indication of the source of the respective strains from the environment (environmental strains) or from patients (clinical strains). Numbers indicated in the table represent strain IDs.

| **Isolates  excluded from phylogeny** | **Corresponding identical isolate included in phylogeny** |
| --- | --- |
| 72 (c), 77 (c), 78 (c), 79 (c) | 80 (c) |
| 67 (c) | 104 (e) |
| 81 (c) | 113 (e) |
| 42 (c), 43 (c), 45 (c), 46 (c), 47 (c), 50 (c) | 85 (e) |
| 39 (c) | 114 (e) |
| 44 (c), 68 (c), 88 (e), 90 (e), 91 (e) | 92 (e) |
| 86 (e) | 115 (e) |
| 69 (c), 87 (e) | 98 (e) |
| 31 (c) | 83 (e) |

*Abbreviations*: e, environmental strain (sampled from environment); c, clinical strain (sampled from patients).

**Supplementary Table 3** Timeline overview (sorted by date of sampling = day of the outbreak analysis) over the course of the outbreak. Patient IDs indicated as ‘0’ constitute environmental samples of different origins.

**a** Cluster 1.

**b** Cluster 2 (early, middle and late time periods of sampling).

**Suppl. Tab. 3 a** **Suppl. Tab. 3 b**

| **Cluster 1** | | |  | **Cluster 2 (day 1-322)** | | |  | **Cluster 2 (day 628-1139)** | | |  | **Cluster 2 (day 1140-1398)** | | |
| --- | --- | --- | --- | --- | --- | --- | --- | --- | --- | --- | --- | --- | --- | --- |
| **Patient ID** | **Strain ID** | **Date of sampling** |  | **Patient ID** | **Strain ID** | **Date of sampling** |  | **Patient ID** | **Strain ID** | **Date of sampling** |  | **Patient ID** | **Strain ID** | **Date of sampling** |
| 1 | 26 | 229 |  | 17 | 57 | 1 |  | 21 | 79 | 628 |  | 0 | 87 | 1140 |
| 2 | 60 | 496 |  | 19 | 72 | 32 |  | 21 | 80 | 640 |  | 0 | 88 | 1140 |
| 2 | 62 | 500 |  | 16 | 51 | 87 |  | 21 | 81 | 642 |  | 11 | 37 | 1145 |
| 2 | 63 | 500 |  | 10 | 30 | 310 |  | 13 | 40 | 697 |  | 0 | 90 | 1147 |
| 2 | 64 | 500 |  | 10 | 29 | 318 |  | 15 | 45 | 962 |  | 0 | 89 | 1151 |
| 2 | 65 | 501 |  | 10 | 31 | 321 |  | 15 | 46 | 964 |  | 14 | 42 | 1153 |
| 2 | 66 | 501 |  | 10 | 32 | 321 |  | 15 | 47 | 964 |  | 14 | 43 | 1155 |
| 3 | 10 | 565 |  | 0 | 83 | 322 |  | 15 | 49 | 983 |  | 0 | 91 | 1156 |
| 3 | 11 | 565 |  |  |  |  |  | 15 | 48 | 985 |  | 0 | 92 | 1156 |
| 3 | 12 | 565 |  |  |  |  |  | 15 | 50 | 992 |  | 0 | 93 | 1165 |
| 3 | 13 | 565 |  |  |  |  |  | 12 | 38 | 1020 |  | 0 | 94 | 1169 |
| 3 | 14 | 565 |  |  |  |  |  | 12 | 39 | 1025 |  | 0 | 96 | 1179 |
| 3 | 6 | 566 |  |  |  |  |  | 0 | 85 | 1033 |  | 0 | 97 | 1182 |
| 3 | 7 | 566 |  |  |  |  |  | 18 | 67 | 1058 |  | 7 | 44 | 1187 |
| 3 | 8 | 566 |  |  |  |  |  | 6 | 68 | 1130 |  | 0 | 98 | 1207 |
| 3 | 9 | 566 |  |  |  |  |  | 20 | 77 | 1131 |  | 5 | 52 | 1214 |
| 3 | 15 | 568 |  |  |  |  |  | 20 | 78 | 1131 |  | 0 | 99 | 1214 |
| 3 | 16 | 572 |  |  |  |  |  | 14 | 41 | 1139 |  | 0 | 100 | 1214 |
| 3 | 17 | 574 |  |  |  |  |  | 0 | 86 | 1139 |  | 0 | 101 | 1214 |
| 3 | 18 | 577 |  |  |  |  |  |  |  |  |  | 0 | 103 | 1221 |
| 3 | 5 | 579 |  |  |  |  |  |  |  |  |  | 0 | 102 | 1222 |
| 3 | 20 | 587 |  |  |  |  |  |  |  |  |  | 0 | 104 | 1228 |
| 3 | 21 | 587 |  |  |  |  |  |  |  |  |  | 0 | 105 | 1228 |
| 3 | 22 | 590 |  |  |  |  |  |  |  |  |  | 6 | 69 | 1235 |
| 3 | 23 | 592 |  |  |  |  |  |  |  |  |  | 6 | 71 | 1293 |
| 3 | 24 | 592 |  |  |  |  |  |  |  |  |  | 0 | 106 | 1326 |
| 3 | 25 | 623 |  |  |  |  |  |  |  |  |  | 0 | 107 | 1389 |
|  |  |  |  |  |  |  |  |  |  |  |  | 0 | 108 | 1396 |
|  |  |  |  |  |  |  |  |  |  |  |  | 0 | 112 | 1398 |
|  |  |  |  |  |  |  |  |  |  |  |  | 0 | 113 | 1398 |
|  |  |  |  |  |  |  |  |  |  |  |  | 0 | 114 | 1398 |
|  |  |  |  |  |  |  |  |  |  |  |  | 0 | 115 | 1398 |

**Supplementary Table 4** Pairwise Distance matrix of the core genomes of all cluster 1 strains plus strain IDs 53, -54 and -55, revealing these three strains (median: 2047) to differ significantly from the 27 strains of cluster 1 (median: 15), as calculated by MEGA7.

| **Strain ID** | 13 | 14 | 15 | 16 | **17** | 18 | 20 | 21 | 22 | 23 | 24 | 25 | **26** | **53** | **54** | **55** | 60 | 62 | 63 | 64 | 65 | 66 | 5 | 6 | 7 | 8 | 9 | 10 | 11 | 12 |
| --- | --- | --- | --- | --- | --- | --- | --- | --- | --- | --- | --- | --- | --- | --- | --- | --- | --- | --- | --- | --- | --- | --- | --- | --- | --- | --- | --- | --- | --- | --- |
| 13 |  |  |  |  |  |  |  |  |  |  |  |  |  |  |  |  |  |  |  |  |  |  |  |  |  |  |  |  |  |  |
| 14 | 9 |  |  |  |  |  |  |  |  |  |  |  |  |  |  |  |  |  |  |  |  |  |  |  |  |  |  |  |  |  |
| 15 | 4 | 7 |  |  |  |  |  |  |  |  |  |  |  |  |  |  |  |  |  |  |  |  |  |  |  |  |  |  |  |  |
| 16 | 26 | 23 | 22 |  |  |  |  |  |  |  |  |  |  |  |  |  |  |  |  |  |  |  |  |  |  |  |  |  |  |  |
| 17 | 18 | 15 | 14 | 12 |  |  |  |  |  |  |  |  |  |  |  |  |  |  |  |  |  |  |  |  |  |  |  |  |  |  |
| 18 | 9 | 10 | 9 | 19 | 15 |  |  |  |  |  |  |  |  |  |  |  |  |  |  |  |  |  |  |  |  |  |  |  |  |  |
| 20 | 13 | 12 | 9 | 15 | 7 | 14 |  |  |  |  |  |  |  |  |  |  |  |  |  |  |  |  |  |  |  |  |  |  |  |  |
| 21 | 35 | 34 | 31 | 21 | 21 | 32 | 24 |  |  |  |  |  |  |  |  |  |  |  |  |  |  |  |  |  |  |  |  |  |  |  |
| 22 | 14 | 13 | 10 | 14 | 4 | 11 | 5 | 23 |  |  |  |  |  |  |  |  |  |  |  |  |  |  |  |  |  |  |  |  |  |  |
| 23 | 29 | 24 | 25 | 25 | 15 | 30 | 16 | 14 | 19 |  |  |  |  |  |  |  |  |  |  |  |  |  |  |  |  |  |  |  |  |  |
| 24 | 19 | 18 | 15 | 11 | 7 | 18 | 12 | 26 | 11 | 18 |  |  |  |  |  |  |  |  |  |  |  |  |  |  |  |  |  |  |  |  |
| 25 | 9 | 6 | 7 | 23 | 11 | 8 | 10 | 32 | 9 | 24 | 18 |  |  |  |  |  |  |  |  |  |  |  |  |  |  |  |  |  |  |  |
| **26** | 17 | 14 | 13 | 13 | **1** | 16 | 6 | 22 | 5 | 14 | 8 | 10 |  |  |  |  |  |  |  |  |  |  |  |  |  |  |  |  |  |  |
| **53** | **2100** | **2105** | **2104** | **2120** | **2114** | **2105** | **2111** | **2133** | **2112** | **2125** | **2111** | **2107** | **2115** |  |  |  |  |  |  |  |  |  |  |  |  |  |  |  |  |  |
| **54** | **2009** | **2012** | **2011** | **2027** | **2023** | **2012** | **2018** | **2040** | **2019** | **2032** | **2018** | **2016** | **2024** | 135 |  |  |  |  |  |  |  |  |  |  |  |  |  |  |  |  |
| **55** | **2047** | **2050** | **2049** | **2065** | **2061** | **2050** | **2058** | **2078** | **2057** | **2072** | **2056** | **2054** | **2062** | 145 | 150 |  |  |  |  |  |  |  |  |  |  |  |  |  |  |  |
| 60 | 11 | 8 | 9 | 19 | 15 | 8 | 8 | 32 | 11 | 22 | 14 | 8 | 14 | **2107** | **2012** | **2052** |  |  |  |  |  |  |  |  |  |  |  |  |  |  |
| 62 | 14 | 9 | 10 | 16 | 10 | 13 | 9 | 27 | 8 | 19 | 11 | 9 | 9 | **2110** | **2017** | **2055** | 7 |  |  |  |  |  |  |  |  |  |  |  |  |  |
| 63 | 21 | 18 | 17 | 7 | 9 | 16 | 10 | 20 | 11 | 18 | 8 | 18 | 8 | **2119** | **2024** | **2062** | 12 | 9 |  |  |  |  |  |  |  |  |  |  |  |  |
| 64 | 15 | 14 | 11 | 15 | 9 | 14 | 4 | 26 | 5 | 18 | 12 | 12 | 8 | **2111** | **2018** | **2058** | 8 | 7 | 10 |  |  |  |  |  |  |  |  |  |  |  |
| 65 | 11 | 8 | 9 | 17 | 9 | 8 | 8 | 28 | 7 | 22 | 12 | 6 | 10 | **2105** | **2014** | **2052** | 6 | 5 | 14 | 8 |  |  |  |  |  |  |  |  |  |  |
| 66 | 9 | 10 | 7 | 21 | 17 | 6 | 14 | 36 | 13 | 30 | 16 | 8 | 16 | **2105** | **2012** | **2050** | 8 | 13 | 18 | 14 | 10 |  |  |  |  |  |  |  |  |  |
| 5 | 15 | 14 | 13 | 17 | 9 | 18 | 6 | 26 | 7 | 16 | 12 | 14 | 8 | **2113** | **2020** | **2058** | 12 | 9 | 10 | 8 | 12 | 18 |  |  |  |  |  |  |  |  |
| 6 | 10 | 9 | 8 | 20 | 18 | 7 | 15 | 37 | 14 | 31 | 17 | 9 | 17 | **2106** | **2013** | **2051** | 9 | 14 | 19 | 15 | 11 | 5 | 19 |  |  |  |  |  |  |  |
| 7 | 12 | 17 | 10 | 16 | 12 | 15 | 11 | 29 | 14 | 21 | 9 | 15 | 11 | **2108** | **2015** | **2055** | 9 | 12 | 11 | 11 | 13 | 13 | 15 | 14 |  |  |  |  |  |  |
| 8 | 16 | 15 | 12 | 12 | 4 | 17 | 5 | 21 | 6 | 15 | 9 | 13 | 3 | **2116** | **2023** | **2061** | 13 | 10 | 7 | 7 | 11 | 17 | 7 | 18 | 10 |  |  |  |  |  |
| 9 | 44 | 49 | 42 | 42 | 36 | 47 | 39 | 55 | 38 | 49 | 35 | 45 | 35 | **2140** | **2049** | **2087** | 45 | 42 | 39 | 39 | 43 | 43 | 39 | 44 | 38 | 36 |  |  |  |  |
| **10** | 16 | 13 | 12 | 12 | 2 | 17 | 5 | 21 | 6 | 13 | 7 | 11 | **1** | **2114** | **2023** | **2061** | 13 | 8 | 7 | 7 | 9 | 17 | 7 | 18 | 10 | 2 | 36 |  |  |  |
| 11 | 7 | 12 | 9 | 27 | 21 | 10 | 18 | 40 | 17 | 34 | 22 | 12 | 20 | **2099** | **2006** | **2044** | 14 | 17 | 24 | 18 | 14 | 10 | 22 | 9 | 15 | 21 | 47 | 21 |  |  |
| 12 | 20 | 19 | 16 | 8 | 8 | 19 | 9 | 17 | 10 | 19 | 13 | 17 | 7 | **2120** | **2027** | **2065** | 17 | 12 | 5 | 11 | 15 | 21 | 11 | 22 | 14 | 6 | 40 | 6 | 25 |  |

**Supplementary Table 5** Cluster 2 accessory genome genes: AG group I, II and III.

**a**, **b**, **c** AG group I; a, gene block 1 (19 genes); b, gene block 2 (10 genes); c, gene block 3 (9 genes).

**d** AG group II, gene block 4 (6 genes).

**e** AG group III, gene block 5 - 10 (total of 31 genes).

UniProt BLAST annotation: the best hit (by identity) is given in the table.

Proteins and/or information mentioned in the text of the manuscript are highlighted in bold.

**Suppl. Tab. 5 a**

| **UniProt BLAST annotation** |  | **GO term** | | | **gene** |
| --- | --- | --- | --- | --- | --- |
| **protein name** | **UniProt acc. no.** | **no.** | **aspect** | **info** | **%GC** |
| **DNA helicase** | [A0A1G5KZ05](http://www.uniprot.org/uniprot/A0A1G5KZ05) | [GO:0005524](https://www.ebi.ac.uk/QuickGO/term/GO:0005524) | F | ATP binding | 57.40 |
|  |  | [GO:0008026](https://www.ebi.ac.uk/QuickGO/term/GO:0008026) | F | **ATP-dependent helicase activity** |  |
|  |  | [GO:0003676](https://www.ebi.ac.uk/QuickGO/term/GO:0003676) | F | nucleic acid binding |  |
| Uncharacterized protein | [A0A1G5L0Q2](http://www.uniprot.org/uniprot/A0A1G5L0Q2) | no GO | - | - | 58.99 |
| **Heat shock protein 70** | [A0A1G5KZ28](http://www.uniprot.org/uniprot/A0A1G5KZ28) | [GO:0005524](https://www.ebi.ac.uk/QuickGO/term/GO:0005524) | F | ATP binding | 57.25 |
| Uncharacterized protein | [A0A1G5KZ52](http://www.uniprot.org/uniprot/A0A1G5KZ52) | no GO | - | - | 58.2 |
| Uncharacterized protein | [A0A1G5KZ60](http://www.uniprot.org/uniprot/A0A1G5KZ60) | [GO:0003676](https://www.ebi.ac.uk/QuickGO/term/GO:0003676) | F | nucleic acid binding | 53.93 |
| Type III restriction-modification system StyLTI enzyme res | [A0A1G5KZ70](http://www.uniprot.org/uniprot/A0A1G5KZ70) | [GO:0005524](https://www.ebi.ac.uk/QuickGO/term/GO:0005524) | F | ATP binding | 58.22 |
|  |  | [GO:0003677](https://www.ebi.ac.uk/QuickGO/term/GO:0003677) | F | DNA binding |  |
|  |  | [GO:0016787](https://www.ebi.ac.uk/QuickGO/term/GO:0016787) | F | hydrolase activity |  |
| Site-specific DNA-methyltransferase (Adenine-specific) | [A0A098FZU8](http://www.uniprot.org/uniprot/A0A098FZU8) | [GO:0009007](https://www.ebi.ac.uk/QuickGO/term/GO:0009007) | F | site-specific DNA-methyltransferase  (adenine-specific) activity | 56.79 |
| Uncharacterized protein | [A0A1G5KZ89](http://www.uniprot.org/uniprot/A0A1G5KZ89) | no GO | - | - | 56.22 |
| **ATP-dependent helicase HepA** | [A0A1G5L015](http://www.uniprot.org/uniprot/A0A1G5L015) | [GO:0005524](https://www.ebi.ac.uk/QuickGO/term/GO:0005524) | F | ATP binding | 56.27 |
|  |  | [GO:0004386](https://www.ebi.ac.uk/QuickGO/term/GO:0004386) | F | **helicase activity** |  |
|  |  | [GO:0016491](https://www.ebi.ac.uk/QuickGO/term/GO:0016491) | F | oxidoreductase activity |  |
| Uncharacterized protein | [A0A1G5KYS8](http://www.uniprot.org/uniprot/A0A1G5KYS8) | no GO | - | - | 54.36 |
| Uncharacterized protein | [A0A1G5KZR3](http://www.uniprot.org/uniprot/A0A1G5KZR3) | [GO:0003824](https://www.ebi.ac.uk/QuickGO/term/GO:0003824) | F | catalytic activity | 56.38 |
| Uncharacterized protein | [A0A1G5KYZ5](http://www.uniprot.org/uniprot/A0A1G5KYZ5) | no GO | - | - | 56.10 |
| Antitoxin | [A0A098FWT3](http://www.uniprot.org/uniprot/A0A098FWT3) | no GO | - | - | 60.44 |
| **DNA repair protein RadC** | [A0A1G5KYU0](http://www.uniprot.org/uniprot/A0A1G5KYU0) | no GO | - | - | 59.43 |
| **Putative phage-type endonuclease** | [A0A1G5KYJ0](http://www.uniprot.org/uniprot/A0A1G5KYJ0) | [GO:0003677](https://www.ebi.ac.uk/QuickGO/term/GO:0003677) | F | DNA binding | 61.39 |
|  |  | [GO:0004519](https://www.ebi.ac.uk/QuickGO/term/GO:0004519) | F | endonuclease activity |  |
| **Phage/plasmid-like protein** | [A0A1G5L075](http://www.uniprot.org/uniprot/A0A1G5L075) | no GO | - | - | 61.09 |
| Uncharacterized protein | [A0A1G5L066](http://www.uniprot.org/uniprot/A0A1G5L066) | no GO | - | - | 41.30 |
| Uncharacterized protein | [A0A1G5KYQ6](http://www.uniprot.org/uniprot/A0A1G5KYQ6) | no GO | - | - | 44.99 |
| **Integrase** | [W8QZ61](http://www.uniprot.org/uniprot/W8QZ61) | [GO:0015074](https://www.ebi.ac.uk/QuickGO/term/GO:0015074) | P | DNA integration | 51.89 |
|  |  | [GO:0006310](https://www.ebi.ac.uk/QuickGO/term/GO:0006310) | P | DNA recombination |  |

**Suppl. Tab. 5 b**

| **UniProt BLAST annotation** |  | **GO term** | | | **gene** |
| --- | --- | --- | --- | --- | --- |
| **protein name** | **UniProt acc. no.** | **nr** | **aspect** | **info** | **%GC** |
| Uncharacterized protein | [A0A0A8RMV6](http://www.uniprot.org/uniprot/A0A0A8RMV6) | [GO:0006260](https://www.ebi.ac.uk/QuickGO/term/GO:0006260) | P | DNA replication | 60.92 |
| Uncharacterized protein | [J9E571](http://www.uniprot.org/uniprot/J9E571) | no GO | - | - | 51.04 |
| Uncharacterized protein | [A0A0A8RRU2](http://www.uniprot.org/uniprot/A0A0A8RRU2) | no GO | - | - | 59.40 |
| Uncharacterized protein | [A0A0A8RSF9](http://www.uniprot.org/uniprot/A0A0A8RSF9) | no GO | - | - | 64.14 |
| Uncharacterized protein | [A0A0A8RN01](http://www.uniprot.org/uniprot/A0A0A8RN01) | no GO | - | - | 61.75 |
| Uncharacterized protein | [W1MPI7](http://www.uniprot.org/uniprot/W1MPI7) | [GO:0016021](https://www.ebi.ac.uk/QuickGO/term/GO:0016021) | C | integral component of membrane | 61.84 |
| Membrane protein | [W1MQ59](http://www.uniprot.org/uniprot/W1MQ59) | [GO:0016021](https://www.ebi.ac.uk/QuickGO/term/GO:0016021) | C | integral component of membrane | 60.61 |
| Uncharacterized protein | [A0A0A8RMV4](http://www.uniprot.org/uniprot/A0A0A8RMV4) | no GO | - | - | 56.93 |
| Uncharacterized protein | [B3G281](http://www.uniprot.org/uniprot/B3G281) | [GO:0016021](https://www.ebi.ac.uk/QuickGO/term/GO:0016021) | C | integral component of membrane | 49.44 |
| Uncharacterized protein | [A0A0H3QL15](http://www.uniprot.org/uniprot/A0A0H3QL15) | [GO:0016021](https://www.ebi.ac.uk/QuickGO/term/GO:0016021) | C | integral component of membrane | 61.69 |

**Suppl. Tab. 5 c**

| **UniProt BLAST annotation** |  | **GO term** | | | **gene** |
| --- | --- | --- | --- | --- | --- |
| **protein name** | **UniProt acc. no.** | **nr** | **aspect** | **info** | **%GC** |
| Uncharacterized protein | [A0A0A8RMV6](http://www.uniprot.org/uniprot/A0A0A8RMV6) | [GO:0006260](https://www.ebi.ac.uk/QuickGO/term/GO:0006260) | P | DNA replication | 60.92 |
| Uncharacterized protein | [J9E571](http://www.uniprot.org/uniprot/J9E571) | no GO | - | - | 51.56 |
| Uncharacterized protein | [A0A0A8RRU2](http://www.uniprot.org/uniprot/A0A0A8RRU2) | no GO | - | - | 59.40 |
| Uncharacterized protein | [A0A0A8RSF9](http://www.uniprot.org/uniprot/A0A0A8RSF9) | no GO | - | - | 64.14 |
| Uncharacterized protein | [W1MPI7](http://www.uniprot.org/uniprot/W1MPI7) | [GO:0016021](https://www.ebi.ac.uk/QuickGO/term/GO:0016021) | C | integral component of membrane | 61.84 |
| Membrane protein | [W1MQ59](http://www.uniprot.org/uniprot/W1MQ59) | [GO:0016021](https://www.ebi.ac.uk/QuickGO/term/GO:0016021) | C | integral component of membrane | 60.61 |
| Uncharacterized protein | [A0A0A8RMV4](http://www.uniprot.org/uniprot/A0A0A8RMV4) | no GO | - | - | 56.93 |
| Uncharacterized protein | [B3G281](http://www.uniprot.org/uniprot/B3G281) | [GO:0016021](https://www.ebi.ac.uk/QuickGO/term/GO:0016021) | C | integral component of membrane | 49.44 |
| Uncharacterized protein | [A0A0H3QL15](http://www.uniprot.org/uniprot/A0A0H3QL15) | [GO:0016021](https://www.ebi.ac.uk/QuickGO/term/GO:0016021) | C | integral component of membrane | 61.69 |

**Suppl. Tab. 5 d**

| **UniProt BLAST annotation** |  | **GO term** | | | **gene** |
| --- | --- | --- | --- | --- | --- |
| **protein name** | **UniProt acc. no.** | **nr** | **aspect** | **info** | **%GC** |
| **Putative integrase** | [A0A220P3T3](http://www.uniprot.org/uniprot/A0A220P3T3) | no GO | - | - | 58.66 |
| Putative transcriptional regulator | [A0A220P3W2](http://www.uniprot.org/uniprot/A0A220P3W2) | no GO | - | - | 54.22 |
| Uncharacterized protein | [A0A220P3R7](http://www.uniprot.org/uniprot/A0A220P3R7) | no GO | - | - | 58.95 |
| Structural protein | [A0A220P3S4](http://www.uniprot.org/uniprot/A0A220P3S4) | no GO | - | - | 57.27 |
| Uncharacterized protein | [A0A220P3Q1](http://www.uniprot.org/uniprot/A0A220P3Q1) | no GO | - | - | 50.23 |
| Uncharacterized protein | [A0A220P3Q4](http://www.uniprot.org/uniprot/A0A220P3Q4) | no GO | - | - | 49.12 |

**Suppl. Tab. 5 e**

| **UniProt BLAST annotation** |  | **GO term** | | | **gene** |
| --- | --- | --- | --- | --- | --- |
| **protein name** | **UniProt acc. no.** | **nr** | **aspect** | **info** | **%GC** |
| RES domain-containing protein | [A0A1H3NHP5](https://www.uniprot.org/uniprot/A0A1H3NHP5) | no GO | - | - | 64.93 |
| Uncharacterized protein | [A0A1H3NHJ4](https://www.uniprot.org/uniprot/A0A1H3NHJ4) | [GO:0016021](https://www.ebi.ac.uk/QuickGO/term/GO:0016021) | C | integral component of membrane | 62.57 |
| Uncharacterized protein | [A0A140S787](https://www.uniprot.org/uniprot/A0A140S787) | [GO:0016021](https://www.ebi.ac.uk/QuickGO/term/GO:0016021) | C | integral component of membrane | 62.00 |
| Uncharacterized protein | [A0A140S9A4](https://www.uniprot.org/uniprot/A0A140S9A4) | [GO:0016021](https://www.ebi.ac.uk/QuickGO/term/GO:0016021) | C | integral component of membrane | 66.38 |
| Uncharacterized protein | [A0A140S7M1](https://www.uniprot.org/uniprot/A0A140S7M1) | no GO | - | - | 68.46 |
| DNA repair protein RadC | [A0A140S6W8](https://www.uniprot.org/uniprot/A0A140S6W8) | [GO:0046872](https://www.ebi.ac.uk/QuickGO/term/GO:0046872) | F | metal ion binding | 59.80 |
|  |  | [GO:0008237](https://www.ebi.ac.uk/QuickGO/term/GO:0008237) | F | metallopeptidase activity |  |
| **Conjugal transfer protein** | [A0A140SAI9](https://www.uniprot.org/uniprot/A0A140SAI9) | no GO | - | - | 64.50 |
| Uncharacterized protein | [A0A140S994](https://www.uniprot.org/uniprot/A0A140S994) | [GO:0016021](https://www.ebi.ac.uk/QuickGO/term/GO:0016021) | C | integral component of membrane | 67.39 |
| Uncharacterized protein | [A0A140S6V8](https://www.uniprot.org/uniprot/A0A140S6V8) | [GO:0016021](https://www.ebi.ac.uk/QuickGO/term/GO:0016021) | C | integral component of membrane | 64.53 |
| **Lytic transglycosylase** | [A0A140S8U1](https://www.uniprot.org/uniprot/A0A140S8U1) | no GO | - | - | 69.64 |
| **Integrating conjugative element protein** | [A0A1Q9R4V8](https://www.uniprot.org/uniprot/A0A1Q9R4V8) | no GO | - | - | 69.28 |
| Uncharacterized protein | [V6ADP2](https://www.uniprot.org/uniprot/V6ADP2) | no GO | - | - | 62.75 |
| Methyltransferase | [A0A140SCL5](https://www.uniprot.org/uniprot/A0A140SCL5) | [GO:0008168](https://www.ebi.ac.uk/QuickGO/term/GO:0008168) | F | methyltransferase activity | 60.44 |
| Uncharacterized protein | [A0A140SCB5](https://www.uniprot.org/uniprot/A0A140SCB5) | no GO | - | - | 66.67 |
| Uncharacterized protein | [A0A140SDV0](https://www.uniprot.org/uniprot/A0A140SDV0) | no GO | - | - | 66.42 |
| Conserved plasmid protein | [A0A140SEF3](https://www.uniprot.org/uniprot/A0A140SEF3) | no GO | - | - | 62.96 |
| Uncharacterized protein | [A0A140SC28](https://www.uniprot.org/uniprot/A0A140SC28) | no GO | - | - | 63.04 |
| GTPase | [A0A140SEM9](https://www.uniprot.org/uniprot/A0A140SEM9) | no GO | - | - | 62.24 |
| Uncharacterized protein | [A0A140SCA4](https://www.uniprot.org/uniprot/A0A140SCA4) | no GO | - | - | 60.55 |
| Uncharacterized protein | [X1LIC0](https://www.uniprot.org/uniprot/X1LIC0) | no GO | - | - | 65.21 |
| Coproporphyrinogen III oxidase | [A0A140SC96](https://www.uniprot.org/uniprot/A0A140SC96) | no GO | - | - | 63.10 |
| Uncharacterized protein | [A0A140SEZ5](https://www.uniprot.org/uniprot/A0A140SEZ5) | no GO | - | - | 66.06 |
| ParA family protein | [A0A2N8G400](https://www.uniprot.org/uniprot/A0A2N8G400) | no GO | - | - | 65.75 |
| **Prophage CP4-57 regulatory protein (AlpA)** | [A0A0N9ZY07](https://www.uniprot.org/uniprot/A0A0N9ZY07) | no GO | - | - | 61.97 |
| Uncharacterized protein | [X1QLC2](https://www.uniprot.org/uniprot/X1QLC2) | no GO | - | - | 52.56 |
| DUF3330 domain-containing protein | [A0A2U2XHT1](https://www.uniprot.org/uniprot/A0A2U2XHT1) | no GO | - | - | 63.53 |
| Uncharacterized protein | [A0A063U963](https://www.uniprot.org/uniprot/A0A063U963) | no GO | - | - | 52.45 |
| Formaldehyde dehydrogenase, glutathione-independent | [A0A1C7BQ95](https://www.uniprot.org/uniprot/A0A1C7BQ95) | [GO:0016491](https://www.ebi.ac.uk/QuickGO/term/GO:0016491) | F | oxidoreductase activity | 55.97 |
|  |  | [GO:0008270](https://www.ebi.ac.uk/QuickGO/term/GO:0008270) | F | zinc ion binding |  |
| Uncharacterized protein | [A0A1C7BZS8](https://www.uniprot.org/uniprot/A0A1C7BZS8) | no GO | - | - | 59.14 |
| Peroxidase | [A0A140SDL1](https://www.uniprot.org/uniprot/A0A140SDL1) | [GO:0020037](https://www.ebi.ac.uk/QuickGO/term/GO:0020037) | F | heme binding | 64.09 |
|  |  | [GO:0004601](https://www.ebi.ac.uk/QuickGO/term/GO:0004601) | F | peroxidase activity |  |
| **AacA4** | [A0A223LNV7](https://www.uniprot.org/uniprot/A0A223LNV7) | no GO | - | - | 54.70 |

*Abbreviations*: GO aspects: B, biological process; C, cellular component; F, molecular function; UniProt acc. no., UniProt accession number; no., number; AG group, accessory genome group.

**Supplementary Table 6** Protein enrichment of different time groups in cluster 2 (unique and shared proteins).

**a** Cluster 2 *early*: unique proteins only part of the early time group of cluster 2 (n=10).

**b** Cluster 2 *late*: unique proteins only part of the late time group of cluster 2 (n=9).

**c** Cluster 2 *early and late*: shared proteins part of both the early and the late time group of cluster 2 (n=6).

UniProt BLAST annotation: the best hit (by identity) is given in the table.

This supplementary table corresponds to Suppl. Fig. 1.

**Suppl. Tab. 6 a**

| **UniProt BLAST annotation** | | **GO term** | | |
| --- | --- | --- | --- | --- |
| **protein name** | **UniProt acc. no.** | **no.** | **aspect** | **info** |
| Bacteriophage replication protein | V1DP08 | - | - | - |
| DNA polymerase III subunit gamma/tau | A0A0H2Z990 | [GO:0009360](https://www.ebi.ac.uk/QuickGO/term/GO:0009360) | C | DNA polymerase III complex |
|  |  | [GO:0005524](https://www.ebi.ac.uk/QuickGO/term/GO:0005524) | F | ATP binding |
|  |  | [GO:0003677](https://www.ebi.ac.uk/QuickGO/term/GO:0003677) | F | ATP binding |
|  |  | [GO:0003887](https://www.ebi.ac.uk/QuickGO/term/GO:0003887) | F | DNA-directed DNA polymerase activity |
|  |  | [GO:0006260](https://www.ebi.ac.uk/QuickGO/term/GO:0006260) | P | DNA replication |
| DNA polymerase III subunits gamma and tau | A0A157WPG4 | - | - | - |
| Dihydroorotase | A0A1G5LWZ4 | [GO:0004151](https://www.ebi.ac.uk/QuickGO/term/GO:0004151) | F | dihydroorotase activity |
|  |  | [GO:0008270](https://www.ebi.ac.uk/QuickGO/term/GO:0008270) | F | zinc ion binding |
|  |  | [GO:0044205](https://www.ebi.ac.uk/QuickGO/term/GO:0044205) | P | de novo' UMP biosynthetic process |
|  |  | [GO:0019856](https://www.ebi.ac.uk/QuickGO/term/GO:0019856) | P | pyrimidine nucleobase biosynthetic process |
| Acetolactate synthase | A1E451 | [GO:0003984](https://www.ebi.ac.uk/QuickGO/term/GO:0003984) | F | acetolactate synthase activity |
|  |  | [GO:0050660](https://www.ebi.ac.uk/QuickGO/term/GO:0050660) | F | flavin adenine dinucleotide binding |
|  |  | [GO:0000287](https://www.ebi.ac.uk/QuickGO/term/GO:0000287) | F | magnesium ion binding |
|  |  | [GO:0030976](https://www.ebi.ac.uk/QuickGO/term/GO:0030976) | F | thiamine pyrophosphate binding |
|  |  | [GO:0009097](https://www.ebi.ac.uk/QuickGO/term/GO:0009097) | P | isoleucine biosynthetic process |
|  |  | [GO:0009099](https://www.ebi.ac.uk/QuickGO/term/GO:0009099) | P | valine biosynthetic process |
| Nucleic acid-binding protein | A0A1F0IY03 | - | - | - |
| SAM-dependent methyltransferase | A0A241X5D3 | [GO:0008168](https://www.ebi.ac.uk/QuickGO/term/GO:0008168) | F | methyltransferase activity |
| Uncharacterized protein | A0A0H3QYR5 | - | - | - |
| Acyl-CoA dehydrogenase, short-chain specific | A0A157WHX2 | [GO:0004085](https://www.ebi.ac.uk/QuickGO/term/GO:0004085) | F | butyryl-CoA dehydrogenase activity |
| Uncharacterized protein | A0A0E9CTQ9 | - | - | - |

**Suppl. Tab. 6 b**

| **UniProt BLAST annotation** | | **GO term** | | |
| --- | --- | --- | --- | --- |
| **protein name** | **UniProt acc. no.** | **no.** | **aspect** | **info** |
| Uncharacterized protein | A0A0H8NS85 | - | - | - |
| **Hcp1 family type VI secretion system effector** | A0A157WVX6 | - | - | - |
| **FHA domain-containing protein** | A0A1S1CAI3 | - | - | - |
| Uncharacterized protein | W1MUL1 | - | - | - |
| Uncharacterized protein | A0A0A8RRG6 | - | - | - |
| Acyltransferase | A0A222CTR8 | [GO:0016747](https://www.ebi.ac.uk/QuickGO/term/GO:0016747) | F | transferase activity, transferring acyl groups other than amino-acyl groups |
| Transposase | A0A0U3JKU6 | [GO:0003677](https://www.ebi.ac.uk/QuickGO/term/GO:0003677) | F | DNA binding |
|  |  | [GO:0004803](https://www.ebi.ac.uk/QuickGO/term/GO:0004803) | F | transposase activity |
|  |  | [GO:0006313](https://www.ebi.ac.uk/QuickGO/term/GO:0006313) | P | transposition, DNA-mediated |
| Transposase | A0A0F7R4E2 | [GO:0003677](https://www.ebi.ac.uk/QuickGO/term/GO:0003677) | F | DNA binding |
|  |  | [GO:0004803](https://www.ebi.ac.uk/QuickGO/term/GO:0004803) | F | transposase activity |
|  |  | [GO:0006313](https://www.ebi.ac.uk/QuickGO/term/GO:0006313) | P | transposition, DNA-mediated |

**Suppl. Tab. 6 c**

| **UniProt BLAST annotation** | | **GO term** | | |
| --- | --- | --- | --- | --- |
| **protein name** | **UniProt acc. no.** | **no.** | **aspect** | **info** |
| **Cold-shock protein** | A0A0V5G2H1 | [GO:0005737](https://www.ebi.ac.uk/QuickGO/term/GO:0005737) | C | cytoplasm |
|  |  | [GO:0003677](https://www.ebi.ac.uk/QuickGO/term/GO:0003677) | F | DNA binding |
|  |  | [GO:0006355](https://www.ebi.ac.uk/QuickGO/term/GO:0006355) | P | regulation of transcription |
|  |  | [GO:0006950](https://www.ebi.ac.uk/QuickGO/term/GO:0006950) | P | response to stress |
| Conjugal transfer protein | A0A1V6KPI1 | - | - | - |
| RAQPRD family plasmid | A0A1V6KP88 | - | - | - |
| Transcriptional regulator | A0A1E9D6X8 | [GO:0003677](https://www.ebi.ac.uk/QuickGO/term/GO:0003677) | F | DNA binding |
|  |  | [GO:0006351](https://www.ebi.ac.uk/QuickGO/term/GO:0006351) | F | DNA binding transcription factor activity |
|  |  | [GO:0006351](https://www.ebi.ac.uk/QuickGO/term/GO:0006351) | P | transcription, DNA-templated |
| Uncharacterized protein | A0A0A8RRB6 | - | - | - |
| **TetR family transcriptional  regulator** | A0A1F0IIG9 | [GO:0003677](https://www.ebi.ac.uk/QuickGO/term/GO:0003677) | F | DNA binding |
|  |  | [GO:0006355](https://www.ebi.ac.uk/QuickGO/term/GO:0006355) | P | regulation of transcription, DNA-templated |
|  |  | [GO:0006351](https://www.ebi.ac.uk/QuickGO/term/GO:0006351) | P | transcription, DNA-templated |

*Abbreviations*: GO aspects: B, biological process; C, cellular component; F, molecular function; UniProt acc. no., UniProt accession number; no., number

**Supplementary Table 7** Functional gene enrichment (GO terms) of different time groups of cluster 2 (n=16). Aspect: B, biological process; C, cellular component; F, molecular function.

| **GO term** | | | **Blast2GO** | |
| --- | --- | --- | --- | --- |
| **nr** | **aspect** | **info** | **FDR** | **P-Value** |
| ***early-intermediate*** | |  |  |  |
| **enriched in early (early > intermediate)** | | |  |  |
| GO:0003995 | F | acyl-CoA dehydrogenase activity | 6,87E-01 | 2,11E-03 |
| GO:0050660 | F | flavin adenine dinucleotide binding | 6,87E-01 | 4,05E-03 |
| GO:0052890 | F | oxidoreductase activity, acting on the CH-CH group of donors, with a flavin as acceptor | 7,94E-01 | 4,83E-02 |
| GO:0004151 | F | dihydroorotase activity | 7,94E-01 | 4,83E-02 |
| GO:0006741 | P | NADP biosynthetic process | 7,94E-01 | 4,83E-02 |
| GO:0006769 | P | nicotinamide metabolic process | 7,94E-01 | 4,83E-02 |
| GO:0046497 | P | nicotinate nucleotide metabolic process | 7,94E-01 | 4,83E-02 |
| GO:0000062 | F | fatty-acyl-CoA binding | 7,94E-01 | 4,83E-02 |
| GO:0019856 | P | pyrimidine nucleobase biosynthetic process | 7,94E-01 | 4,83E-02 |
| GO:0044205 | P | 'de novo' UMP biosynthetic process | 7,94E-01 | 4,83E-02 |
| GO:0003951 | F | NAD+ kinase activity | 7,94E-01 | 4,83E-02 |
| GO:0055088 | P | lipid homeostasis | 7,94E-01 | 4,83E-02 |
| GO:0033539 | P | fatty acid beta-oxidation using acyl-CoA dehydrogenase | 7,94E-01 | 4,83E-02 |
| ***intermediate-late*** | |  |  |  |
| **enriched in late (intermediate < late)** | | |  |  |
| GO:0006310 | P | DNA recombination | 1,00E+00 | 1,82E-02 |
| GO:0051346 | P | negative regulation of hydrolase activity | 1,00E+00 | 2,97E-02 |
| GO:0003677 | F | DNA binding | 1,00E+00 | 3,40E-02 |
| ***early-late*** |  |  |  |  |
| no enrichment |  |  |  |  |

*Abbreviation*: FDR, false discovery rate.

**Supplementary Table 8** Genome and assembly statistics, plus data on contamination and genome completeness calculated using CheckM, and indication of ENA accession numbers.

| **Strain ID** | **Genome size** | **Contigs** | **N50** | **%GC** | **contami- nation (%)** | **genome  complete- ness (%)** | **ENA 1° Accession** | **ENA 2° Accession** |
| --- | --- | --- | --- | --- | --- | --- | --- | --- |
| 1 | 6772897 | 127 | 202539 | 65.76 | 0.14 | 99.68 | ERS2878687 | SAMEA5067485 |
| 2 | 7053529 | 101 | 189319 | 65.8 | 0.11 | 99.68 | ERS2878688 | SAMEA5067486 |
| 3 | 7072906 | 153 | 170981 | 65.78 | 0.11 | 99.68 | ERS2878689 | SAMEA5067487 |
| 5 | 7426251 | 139 | 177606 | 65.25 | 1.35 | 99.68 | ERS2878690 | SAMEA5067488 |
| 6 | 7040054 | 121 | 223784 | 65.72 | 0.11 | 99.68 | ERS2878691 | SAMEA5067489 |
| 7 | 7042309 | 123 | 212129 | 65.72 | 0.11 | 99.68 | ERS2878692 | SAMEA5067490 |
| 8 | 7041566 | 131 | 188546 | 65.72 | 0.11 | 99.68 | ERS2878693 | SAMEA5067491 |
| 9 | 7037407 | 139 | 212129 | 65.72 | 0.11 | 99.68 | ERS2878694 | SAMEA5067492 |
| 10 | 7042973 | 136 | 163528 | 65.72 | 0.11 | 99.68 | ERS2878695 | SAMEA5067493 |
| 11 | 7040074 | 118 | 174017 | 65.72 | 0.11 | 99.68 | ERS2878696 | SAMEA5067494 |
| 12 | 7041515 | 123 | 223784 | 65.72 | 0.11 | 99.68 | ERS2878697 | SAMEA5067495 |
| 13 | 7043666 | 127 | 212129 | 65.72 | 0.11 | 99.68 | ERS2878698 | SAMEA5067496 |
| 14 | 7041600 | 142 | 173924 | 65.72 | 0.11 | 99.59 | ERS2878699 | SAMEA5067497 |
| 15 | 7041103 | 138 | 188557 | 65.72 | 0.13 | 99.68 | ERS2878700 | SAMEA5067498 |
| 16 | 7042147 | 144 | 154725 | 65.72 | 0.27 | 99.68 | ERS2878701 | SAMEA5067499 |
| 17 | 7041043 | 129 | 177606 | 65.72 | 2.71 | 99.68 | ERS2878702 | SAMEA5067500 |
| 18 | 7424332 | 168 | 129713 | 65.25 | 1.35 | 99.68 | ERS2878703 | SAMEA5067501 |
| 19 | 6766231 | 129 | 138396 | 66.1 | 0.14 | 99.68 | ERS2878704 | SAMEA5067502 |
| 20 | 7042955 | 139 | 161870 | 65.72 | 0.11 | 99.68 | ERS2878705 | SAMEA5067503 |
| 21 | 7039982 | 135 | 173924 | 65.72 | 0.11 | 99.68 | ERS2878706 | SAMEA5067504 |
| 22 | 7046523 | 150 | 167623 | 65.72 | 0.11 | 99.68 | ERS2878707 | SAMEA5067505 |
| 23 | 7045447 | 139 | 183315 | 65.72 | 0.11 | 99.68 | ERS2878708 | SAMEA5067506 |
| 24 | 7043421 | 124 | 173924 | 65.72 | 0.11 | 99.68 | ERS2878709 | SAMEA5067507 |
| 25 | 7042411 | 130 | 216284 | 65.72 | 0.11 | 99.68 | ERS2878710 | SAMEA5067508 |
| 26 | 7045396 | 144 | 177606 | 65.72 | 0.11 | 99.68 | ERS2878711 | SAMEA5067509 |
| 28 | 6953041 | 108 | 227865 | 65.94 | 0.74 | 99.68 | ERS2878712 | SAMEA5067510 |
| 29 | 7178268 | 138 | 164738 | 65.7 | 0.84 | 99.68 | ERS2878713 | SAMEA5067511 |
| 30 | 7176971 | 134 | 154950 | 65.71 | 0.84 | 99.68 | ERS2878714 | SAMEA5067512 |
| 31 | 7177252 | 133 | 231204 | 65.71 | 0.84 | 99.68 | ERS2878715 | SAMEA5067513 |
| 32 | 7177530 | 123 | 222806 | 65.7 | 0.84 | 99.68 | ERS2878716 | SAMEA5067514 |
| 35 | 6988208 | 219 | 163293 | 65.92 | 0.53 | 99.27 | ERS2878717 | SAMEA5067515 |
| 36 | 6975458 | 165 | 167108 | 65.92 | 0.53 | 99.35 | ERS2878718 | SAMEA5067516 |
| 37 | 7182072 | 134 | 231567 | 65.7 | 0.84 | 99.68 | ERS2878719 | SAMEA5067517 |
| 38 | 7177254 | 127 | 202440 | 65.71 | 0.84 | 99.68 | ERS2878720 | SAMEA5067518 |
| 39 | 7176685 | 143 | 164650 | 65.71 | 0.84 | 99.68 | ERS2878721 | SAMEA5067519 |
| 40 | 7175399 | 130 | 191588 | 65.71 | 0.84 | 99.68 | ERS2878722 | SAMEA5067520 |
| 41 | 7213094 | 191 | 154951 | 65.69 | 1.16 | 99.68 | ERS2878723 | SAMEA5067521 |
| 42 | 7176316 | 137 | 191588 | 65.7 | 0.84 | 99.68 | ERS2878724 | SAMEA5067522 |
| 43 | 7177461 | 139 | 207130 | 65.71 | 0.84 | 99.68 | ERS2878725 | SAMEA5067523 |
| 44 | 7170318 | 128 | 207136 | 65.72 | 0.84 | 99.68 | ERS2878726 | SAMEA5067524 |
| 45 | 7178699 | 143 | 216372 | 65.7 | 0.84 | 99.35 | ERS2878727 | SAMEA5067525 |
| 46 | 7251572 | 163 | 147072 | 65.67 | 1.16 | 99.68 | ERS2878728 | SAMEA5067526 |
| 47 | 7230002 | 190 | 164447 | 65.68 | 1.16 | 99.68 | ERS2878729 | SAMEA5067527 |
| 48 | 7248455 | 138 | 178145 | 65.68 | 1.16 | 99.68 | ERS2878730 | SAMEA5067528 |
| 49 | 7244131 | 156 | 168590 | 65.68 | 1.16 | 99.68 | ERS2878731 | SAMEA5067529 |
| 50 | 7179347 | 136 | 202272 | 65.7 | 0.87 | 99.68 | ERS2878732 | SAMEA5067530 |
| 51 | 7177444 | 132 | 167292 | 65.71 | 0.84 | 99.59 | ERS2878733 | SAMEA5067531 |
| 52 | 7159137 | 138 | 210805 | 65.74 | 0.84 | 99.68 | ERS2878734 | SAMEA5067532 |
| 53 | 7098673 | 110 | 174017 | 65.79 | 0.11 | 99.68 | ERS2878735 | SAMEA5067533 |
| 54 | 7096213 | 128 | 159981 | 65.79 | 0.11 | 99.68 | ERS2878736 | SAMEA5067534 |
| 55 | 7100349 | 112 | 180036 | 65.79 | 0.11 | 99.68 | ERS2878737 | SAMEA5067535 |
| 57 | 7177899 | 131 | 192788 | 65.7 | 0.84 | 99.68 | ERS2878738 | SAMEA5067536 |
| 58 | 6895720 | 70 | 231054 | 65.81 | 1.35 | 99.68 | ERS2878739 | SAMEA5067537 |
| 59 | 6925743 | 125 | 344875 | 65.77 | 1.52 | 99.68 | ERS2878740 | SAMEA5067538 |
| 60 | 7101206 | 347 | 144958 | 65.68 | 0.11 | 99.59 | ERS2878741 | SAMEA5067539 |
| 61 | 6904567 | 98 | 225935 | 65.78 | 1.35 | 99.68 | ERS2878742 | SAMEA5067540 |
| 62 | 7305207 | 455 | 194183 | 65.4 | 1.19 | 99.59 | ERS2878743 | SAMEA5067541 |
| 63 | 7409612 | 295 | 141429 | 65.27 | 1.52 | 99.68 | ERS2878744 | SAMEA5067542 |
| 64 | 7161952 | 343 | 147749 | 65.57 | 0.22 | 99.68 | ERS2878745 | SAMEA5067543 |
| 65 | 7346097 | 413 | 173924 | 65.34 | 1.41 | 99.68 | ERS2878746 | SAMEA5067544 |
| 66 | 7437299 | 196 | 189592 | 65.23 | 1.35 | 99.59 | ERS2878747 | SAMEA5067545 |
| 67 | 7178085 | 136 | 192793 | 65.7 | 0.84 | 99.68 | ERS2878748 | SAMEA5067546 |
| 68 | 7174430 | 143 | 191588 | 65.71 | 0.84 | 99.59 | ERS2878749 | SAMEA5067547 |
| 69 | 7181806 | 147 | 173161 | 65.69 | 0.84 | 99.68 | ERS2878750 | SAMEA5067548 |
| 71 | 7203107 | 182 | 216372 | 65.63 | 1.21 | 99.68 | ERS2878751 | SAMEA5067549 |
| 72 | 7179909 | 134 | 199969 | 65.7 | 0.84 | 99.68 | ERS2878752 | SAMEA5067550 |
| 76 | 6362618 | 211 | 211049 | 66.41 | 0.11 | 99.68 | ERS2878753 | SAMEA5067551 |
| 77 | 7185649 | 150 | 199983 | 65.68 | 0.84 | 99.68 | ERS2878754 | SAMEA5067552 |
| 78 | 7181449 | 148 | 199701 | 65.7 | 0.84 | 99.68 | ERS2878755 | SAMEA5067553 |
| 79 | 7175542 | 158 | 154947 | 65.71 | 0.84 | 99.68 | ERS2878756 | SAMEA5067554 |
| 80 | 7180488 | 154 | 155360 | 65.7 | 0.84 | 99.68 | ERS2878757 | SAMEA5067555 |
| 81 | 7182071 | 151 | 192775 | 65.7 | 0.84 | 99.68 | ERS2878758 | SAMEA5067556 |
| 83 | 7186905 | 163 | 184431 | 65.7 | 0.84 | 99.68 | ERS2878759 | SAMEA5067557 |
| 85 | 7236482 | 437 | 126104 | 65.71 | 10.25 | 99.68 | ERS2878760 | SAMEA5067558 |
| 86 | 7176975 | 131 | 164440 | 65.71 | 0.84 | 99.68 | ERS2878761 | SAMEA5067559 |
| 87 | 7181179 | 148 | 191588 | 65.7 | 0.84 | 99.59 | ERS2878762 | SAMEA5067560 |
| 88 | 7171955 | 145 | 202440 | 65.72 | 0.84 | 99.59 | ERS2878763 | SAMEA5067561 |
| 89 | 7179328 | 140 | 191588 | 65.7 | 0.84 | 99.68 | ERS2878764 | SAMEA5067562 |
| 90 | 7173438 | 133 | 222806 | 65.72 | 0.84 | 99.68 | ERS2878765 | SAMEA5067563 |
| 91 | 7172169 | 137 | 214702 | 65.72 | 0.84 | 99.68 | ERS2878766 | SAMEA5067564 |
| 92 | 7169893 | 143 | 154950 | 65.72 | 0.84 | 99.68 | ERS2878767 | SAMEA5067565 |
| 93 | 7157357 | 144 | 191588 | 65.74 | 0.84 | 99.68 | ERS2878768 | SAMEA5067566 |
| 94 | 7167337 | 200 | 155549 | 65.74 | 0.84 | 99.68 | ERS2878769 | SAMEA5067567 |
| 96 | 7220347 | 299 | 146553 | 65.68 | 3.11 | 99.68 | ERS2878770 | SAMEA5067568 |
| 97 | 7153774 | 157 | 147082 | 65.75 | 0.84 | 99.68 | ERS2878771 | SAMEA5067569 |
| 98 | 7180709 | 162 | 154948 | 65.7 | 0.84 | 99.68 | ERS2878772 | SAMEA5067570 |
| 99 | 7154826 | 163 | 187032 | 65.74 | 0.84 | 99.59 | ERS2878773 | SAMEA5067571 |
| 100 | 7147689 | 142 | 154945 | 65.75 | 0.84 | 99.68 | ERS2878774 | SAMEA5067572 |
| 101 | 7158652 | 144 | 155466 | 65.74 | 0.84 | 99.68 | ERS2878775 | SAMEA5067573 |
| 102 | 7158569 | 128 | 222806 | 65.74 | 0.84 | 99.68 | ERS2878776 | SAMEA5067574 |
| 103 | 7153130 | 127 | 222806 | 65.75 | 0.84 | 99.68 | ERS2878777 | SAMEA5067575 |
| 104 | 7177762 | 117 | 233089 | 65.71 | 0.84 | 99.68 | ERS2878778 | SAMEA5067576 |
| 105 | 7155366 | 123 | 216372 | 65.75 | 0.84 | 99.68 | ERS2878779 | SAMEA5067577 |
| 106 | 7155899 | 121 | 231208 | 65.74 | 0.84 | 99.68 | ERS2878780 | SAMEA5067578 |
| 107 | 7177164 | 123 | 231567 | 65.71 | 0.84 | 99.68 | ERS2878781 | SAMEA5067579 |
| 108 | 7164940 | 121 | 218222 | 65.71 | 0.84 | 99.68 | ERS2878782 | SAMEA5067580 |
| 112 | 7146500 | 106 | 227638 | 65.74 | 0.51 | 99.68 | ERS2878783 | SAMEA5067581 |
| 113 | 7180623 | 126 | 233089 | 65.7 | 0.84 | 99.68 | ERS2878784 | SAMEA5067582 |
| 114 | 7177248 | 107 | 268317 | 65.71 | 0.84 | 99.68 | ERS2878785 | SAMEA5067583 |
| 115 | 7179043 | 119 | 231204 | 65.71 | 0.84 | 99.68 | ERS2878786 | SAMEA5067584 |

*Abbreviations*: ENA 1° Accession, ENA primary accession number; ENA 2° Accession, ENA secondary accession number.

**Supplementary Table 9** Overview of antibiotic susceptibility patterns and known resistance genes of all strains.

**a**, Phenotypic resistance (determined by broth microdilution antimicrobial susceptibility testing).

**b**, Genotypic resistance (calculated using ResFinder).

**Suppl. Tab. 9 a**

| **Phenotypic resistance** | | | |  |  |  |  |  |  |  |  |  |  |
| --- | --- | --- | --- | --- | --- | --- | --- | --- | --- | --- | --- | --- | --- |
| **Strain ID** | **GEN** | **TOB** | **PIP** | **PIT** | **CTZ** | **LEV** | **CIP** | **MER** | **CEP** | **FOS** | **AZT** | **COL** | **AMI** |
| 1 | R | R | R | S | R | R | R | R | R | R | I | S | S |
| 2 | R | R | R | R | R | R | R | R | R | R | R | S | R |
| 3 | R | R | R | R | R | R | R | R | R | R | I | S | S |
| 5 | R | R | R | R | R | R | R | R | R | S | R | S | R |
| 6 | R | R | R | R | S | R | R | R | S | NB | I | S | S |
| 7 | R | R | R | R | S | R | R | R | S | I | I | S | R |
| 8 | R | R | R | R | S | R | R | R | S | S | I | S | S |
| 9 | R | R | R | R | R | R | R | R | S | - | - | - | - |
| 10 | R | R | R | R | R | R | R | R | S | - | - | - | - |
| 11 | R | R | R | R | R | R | R | R | S | S | I | S | S |
| 12 | R | R | R | R | S | R | R | R | S | S | I | S | S |
| 13 | R | R | R | R | S | R | R | R | S | - | - | - | - |
| 14 | R | R | R | R | S | R | R | R | S | S | I | S | S |
| 15 | R | R | R | R | S | R | R | R | S | S | I | S | S |
| 16 | R | R | R | R | S | R | R | R | S | S | I | S | S |
| 17 | R | R | R | R | S | R | R | R | S | S | I | S | S |
| 18 | R | R | R | R | R | R | R | R | R | S | I | S | R |
| 19 | R | R | R | R | S | R | R | R | S | S | R | S | R |
| 20 | R | R | R | R | S | R | R | R | S | S | R | S | S |
| 21 | R | R | R | R | S | R | R | R | S | S | I | S | S |
| 22 | R | R | R | R | S | R | R | R | S | S | I | S | S |
| 23 | R | R | R | R | I | R | R | R | S | S | I | S | S |
| 24 | R | R | R | R | S | R | R | R | S | S | I | S | S |
| 25 | R | R | R | R | R | R | R | R | S | S | I | S | S |
| 26 | R | R | R | R | R | R | R | R | R | R | R | S | R |
| 28 | S | S | S | S | S | R | I | S | S | R | R | S | R |
| 29 | R | R | R | R | R | R | R | R | R | R | I | S | R |
| 30 | R | R | R | R | R | R | R | R | R | R | I | S | R |
| 31 | R | R | R | R | R | R | R | R | R | R | I | S | R |
| 32 | R | R | R | R | R | R | R | R | R | R | I | S | R |
| 35 | R | R | R | R | R | R | R | R | R | I | I | S | S |
| 36 | R | R | R | R | R | R | R | R | R | R | I | S | S |
| 37 | R | R | R | R | R | R | R | R | R | R | I | S | R |
| 38 | R | R | R | R | R | R | R | R | R | NB | I | S | R |
| 39 | R | R | R | R | R | R | R | R | R | R | R | S | R |
| 40 | R | R | R | R | R | R | R | R | R | R | I | S | R |
| 41 | R | R | R | R | R | R | R | R | R | R | R | S | R |
| 42 | R | R | R | R | R | R | R | R | R | R | R | S | R |
| 43 | R | R | R | R | R | R | R | R | R | R | R | S | R |
| 44 | R | R | R | R | R | R | R | R | R | NB | I | S | R |
| 45 | R | R | R | R | R | R | R | R | R | R | R | S | R |
| 46 | R | R | R | R | R | R | R | R | R | R | R | S | R |
| 47 | R | R | R | R | R | R | R | R | R | R | R | S | R |
| 48 | R | R | R | R | R | R | R | R | R | R | R | S | R |
| 49 | R | R | R | R | R | R | R | R | R | R | R | S | R |
| 50 | R | R | R | R | R | R | R | R | R | R | R | S | R |
| 51 | R | R | R | R | R | R | R | R | R | R | R | S | R |
| 52 | R | R | R | R | R | R | R | R | R | R | I | S | R |
| 53 | R | R | R | R | R | R | R | R | R | NB | I | S | R |
| 54 | R | R | R | R | R | R | R | R | R | R | R | S | R |
| 55 | R | R | R | R | R | R | R | I | R | NB | I | S | R |
| 57 | R | R | R | R | R | R | R | R | R | R | R | S | R |
| 58 | R | R | R | R | R | S | S | R | S | I | I | S | I |
| 59 | R | R | R | R | R | R | R | R | R | R | I | S | R |
| 60 | R | R | R | R | S | R | R | R | S | R | I | S | R |
| 61 | R | R | R | R | S | R | R | R | S | R | I | S | R |
| 62 | R | R | R | R | R | R | R | R | R | S | R | S | R |
| 63 | R | R | R | R | R | R | R | R | R | S | R | S | R |
| 64 | R | R | R | R | R | R | R | R | R | S | R | S | R |
| 65 | R | R | R | R | S | R | R | R | R | S | R | R | R |
| 66 | R | R | R | R | R | R | R | R | R | S | R | S | R |
| 67 | R | R | R | R | R | R | R | R | R | R | I | S | R |
| 68 | R | R | R | R | R | R | R | R | R | R | I | S | R |
| 69 | R | R | R | R | R | R | R | R | R | R | R | S | R |
| 71 | R | R | R | R | R | R | R | R | R | R | R | S | R |
| 72 | R | R | R | R | R | R | R | R | R | R | R | S | R |
| 76 | R | R | R | R | R | R | R | R | R | R | R | S | R |
| 77 | R | R | R | R | R | R | R | R | R | R | R | S | R |
| 78 | R | R | R | R | R | R | R | R | R | R | R | S | R |
| 79 | R | R | R | R | R | R | R | R | R | R | R | S | R |
| 80 | R | R | R | R | R | R | R | R | R | R | R | S | R |
| 81 | R | R | R | R | R | R | R | R | R | R | R | S | R |
| 83 | R | R | R | R | R | R | R | R | R | R | R | S | R |
| 85 | R | R | R | R | R | R | R | R | R | R | R | S | R |
| 86 | R | R | R | R | R | R | R | R | R | R | R | S | R |
| 87 | R | R | R | R | R | R | R | R | R | R | R | S | R |
| 88 | R | R | R | R | R | R | R | R | R | R | R | S | R |
| 89 | R | R | R | R | R | R | R | R | R | R | R | S | R |
| 90 | R | R | R | R | R | R | R | R | R | R | R | S | R |
| 91 | R | R | R | R | R | R | R | R | R | R | R | S | R |
| 92 | R | R | R | R | R | R | R | R | R | R | R | S | R |
| 93 | R | R | R | R | R | R | R | R | R | R | R | S | R |
| 94 | R | R | R | R | R | R | R | R | R | R | R | S | R |
| 96 | R | R | R | R | R | R | R | R | R | R | R | S | R |
| 97 | R | R | R | R | R | R | R | R | R | R | R | S | R |
| 98 | R | R | R | R | R | R | R | R | R | R | R | S | R |
| 99 | R | R | R | R | R | R | R | R | R | R | R | S | R |
| 100 | R | R | R | R | R | R | R | R | R | R | R | S | R |
| 101 | R | R | R | R | R | R | R | R | R | R | R | S | R |
| 102 | R | R | R | R | R | R | R | R | R | R | R | S | R |
| 103 | R | R | R | R | R | R | R | R | R | R | R | S | R |
| 104 | R | R | R | R | R | R | R | R | R | R | R | S | R |
| 105 | R | R | R | R | R | R | R | R | R | R | R | S | R |
| 106 | R | R | R | R | R | R | R | R | R | R | R | S | R |
| 107 | R | R | R | R | R | R | R | R | R | R | R | S | R |
| 108 | R | R | R | R | R | R | R | R | R | R | R | S | R |
| 112 | R | R | R | R | R | R | R | R | R | R | R | S | R |
| 113 | R | R | R | R | R | R | R | R | R | R | R | S | R |
| 114 | R | R | R | R | R | R | R | R | R | R | R | S | R |
| 115 | R | R | R | R | R | R | R | R | R | R | R | S | R |

*Abbreviations*: GEN, gentamicin; TOB, tobramycin; PIP, piperacillin; PIT, piperacillin-tazobactam; CTZ, ceftazidime; LEV, levofloxacin; CIP, ciprofloxacin; MER, meropenem; CEP, cefepime; FOS, fosfomycin; AZT, aztreonam; COL, colistin; AMI, amikacin.

R, resistant; S, susceptible; I, intermediate; NB, undeterminate; -, no data.

**Suppl. Tab. 9 b**

| **Genotypic resistance** | |  |  |  |  |  |  |  |  |
| --- | --- | --- | --- | --- | --- | --- | --- | --- | --- |
| **Strain ID** | **Aminoglycoside** | **Fosfomycin** | **Sulphonamide** | **Trimethoprim** | **Tetracycline** | **Phenicol** | **Beta-lactam** | **Integron** | **Fluoroquinolone and aminoglycoside** |
| 1 | aac(6')-IIc, aph(3')-IIb-like | fosA-like | sul1 |  |  | catB7-like | blaOXA-50-like, blaPAO-like, blaVIM-2 |  |  |
| 2 | aac(6')-IIc, aph(3')-IIb-like | fosA-like | sul1 |  |  | catB7-like | blaOXA-50-like, blaPAO-like, blaVIM-2 |  |  |
| 3 | aac(6')-IIc, aph(3')-IIb-like | fosA-like | sul1 |  |  | catB7-like | blaOXA-50-like, blaPAO-like, blaVIM-2 |  |  |
| 5 | aacA29-like, aadB | fosA-like | sul1 |  |  | catB7-like | blaOXA-10, blaOXA-50, blaPAO-like, blaVIM-2 | aacA4-like | aac(6')Ib-cr-like |
| 6 |  | fosA-like | sul1 |  |  | catB7-like | blaOXA-50, blaPAO-like | aacA4-like | aac(6')Ib-cr-like |
| 7 |  | fosA-like | sul1 |  |  | catB7-like | blaOXA-50, blaPAO-like | aacA4-like | aac(6')Ib-cr-like |
| 8 |  | fosA-like | sul1 |  |  | catB7-like | blaOXA-50, blaPAO-like | aacA4-like | aac(6')Ib-cr-like |
| 9 |  | fosA-like | sul1 |  |  | catB7-like | blaOXA-50, blaPAO-like | aacA4-like | aac(6')Ib-cr-like |
| 10 |  | fosA-like | sul1 |  |  | catB7-like | blaOXA-50, blaPAO-like | aacA4-like | aac(6')Ib-cr-like |
| 11 |  | fosA-like | sul1 |  |  | catB7-like | blaOXA-50, blaPAO-like | aacA4-like | aac(6')Ib-cr-like |
| 12 |  | fosA-like | sul1 |  |  | catB7-like | blaOXA-50, blaPAO-like | aacA4-like | aac(6')Ib-cr-like |
| 13 |  | fosA-like | sul1 |  |  | catB7-like | blaOXA-50, blaPAO-like | aacA4-like | aac(6')Ib-cr-like |
| 14 |  | fosA-like | sul1 |  |  | catB7-like | blaOXA-50, blaPAO-like | aacA4-like | aac(6')Ib-cr-like |
| 15 |  | fosA-like | sul1 |  |  | catB7-like | blaOXA-50, blaPAO-like | aacA4-like | aac(6')Ib-cr-like |
| 16 |  | fosA-like | sul1 |  |  | catB7-like | blaOXA-50, blaPAO-like | aacA4-like | aac(6')Ib-cr-like |
| 17 |  | fosA-like | sul1 |  |  | catB7-like | blaOXA-50, blaPAO-like | aacA4-like | aac(6')Ib-cr-like |
| 18 | aacA29-like, aadB | fosA-like | sul1 |  |  | catB7-like | blaOXA-10, blaOXA-50, blaPAO-like, blaVIM-2 | aacA4-like | aac(6')Ib-cr-like |
| 19 | aph(3')-IIb-like | fosA-like |  |  |  | catB7-like | blaOXA-50-like, blaPAO-like |  |  |
| 20 |  | fosA-like | sul1 |  |  | catB7-like | blaOXA-50, blaPAO-like | aacA4-like | aac(6')Ib-cr-like |
| 21 |  | fosA-like | sul1 |  |  | catB7-like | blaOXA-50, blaPAO-like | aacA4-like | aac(6')Ib-cr-like |
| 22 |  | fosA-like | sul1 |  |  | catB7-like | blaOXA-50, blaPAO-like | aacA4-like | aac(6')Ib-cr-like |
| 23 |  | fosA-like | sul1 |  |  | catB7-like | blaOXA-50, blaPAO-like | aacA4-like | aac(6')Ib-cr-like |
| 24 |  | fosA-like | sul1 |  |  | catB7-like | blaOXA-50, blaPAO-like | aacA4-like | aac(6')Ib-cr-like |
| 25 |  | fosA-like | sul1 |  |  | catB7-like | blaOXA-50, blaPAO-like | aacA4-like | aac(6')Ib-cr-like |
| 26 |  | fosA-like | sul1 |  |  | catB7-like | blaOXA-50, blaPAO-like | aacA4-like | aac(6')Ib-cr-like |
| 28 | aph(3')-IIb-like | fosA-like |  |  |  | catB7-like | blaOXA-50, blaPAO-like |  |  |
| 29 | aac(3)-Id-like, aadA2-like, aph(3')-IIb-like | fosA-like | sul1 | dfrB5 | tet(G) | catB7-like, cmlA1-like | blaOXA-4, blaOXA-50-like, blaPAO-like, blaVIM-2 |  |  |
| 30 | aac(3)-Id-like, aadA2-like, aph(3')-IIb-like | fosA-like | sul1-like | dfrB5 | tet(G) | catB7-like, cmlA1-like | blaOXA-4, blaOXA-50-like, blaPAO-like, blaVIM-2 |  |  |
| 31 | aac(3)-Id-like, aadA2-like, aph(3')-IIb-like | fosA-like | sul1 | dfrB5 | tet(G) | catB7-like, cmlA1-like | blaOXA-4, blaOXA-50-like, blaPAO-like, blaVIM-2 |  |  |
| 32 | aac(3)-Id-like, aadA2-like, aph(3')-IIb-like | fosA-like | sul1 | dfrB5 | tet(G) | catB7-like, cmlA1-like | blaOXA-4, blaOXA-50-like, blaPAO-like, blaVIM-2 |  |  |
| 35 | aac(3)-Ic, aadB, aph(3')-IIb-like, strA, strB | fosA-like | sul1 |  |  | catB7-like, cmlA1-like | blaOXA-10, blaOXA-50-like, blaPAO-like, blaVIM-2 |  |  |
| 36 | aac(3)-Ic, aadB, aph(3')-IIb-like, strA, strB | fosA-like | sul1 |  |  | catB7-like, cmlA1-like | blaOXA-10, blaOXA-50-like, blaPAO-like, blaVIM-2 |  |  |
| 37 | aac(3)-Id-like, aadA2-like, aph(3')-IIb-like | fosA-like | sul1-like | dfrB5 | tet(G) | catB7-like, cmlA1-like | blaOXA-4, blaOXA-50-like, blaPAO-like, blaVIM-2 |  |  |
| 38 | aac(3)-Id-like, aadA2-like, aph(3')-IIb-like | fosA-like | sul1 | dfrB5 | tet(G) | catB7-like, cmlA1-like | blaOXA-4, blaOXA-50-like, blaPAO-like, blaVIM-2 |  |  |
| 39 | aac(3)-Id-like, aadA2-like, aph(3')-IIb-like | fosA-like | sul1 | dfrB5 | tet(G) | catB7-like, cmlA1-like | blaOXA-4, blaOXA-50-like, blaPAO-like, blaVIM-2 |  |  |
| 40 | aac(3)-Id-like, aadA2-like, aph(3')-IIb-like | fosA-like | sul1-like | dfrB5 | tet(G) | catB7-like, cmlA1-like | blaOXA-4, blaOXA-50-like, blaPAO-like, blaVIM-2 |  |  |
| 41 | aac(3)-Id-like, aadA2-like, aph(3')-IIb-like | fosA-like | sul1 | dfrB5 | tet(G) | catB7-like, cmlA1-like | blaOXA-4, blaOXA-50-like, blaPAO-like, blaVIM-2 | aacA4-like | aac(6')Ib-cr-like |
| 42 | aac(3)-Id-like, aadA2-like, aph(3')-IIb-like | fosA-like | sul1-like | dfrB5 | tet(G) | catB7-like, cmlA1-like | blaOXA-4, blaOXA-50-like, blaPAO-like, blaVIM-2 |  |  |
| 43 | aac(3)-Id-like, aadA2-like, aph(3')-IIb-like | fosA-like | sul1 | dfrB5 | tet(G) | catB7-like, cmlA1-like | blaOXA-4, blaOXA-50-like, blaPAO-like, blaVIM-2 |  |  |
| 44 | aac(3)-Id-like, aadA2-like, aph(3')-IIb-like | fosA-like | sul1-like | dfrB5 | tet(G) | catB7-like, cmlA1-like | blaOXA-4, blaOXA-50-like, blaPAO-like, blaVIM-2 |  |  |
| 45 | aac(3)-Id-like, aadA2-like, aph(3')-IIb-like | fosA-like | sul1 | dfrB5 | tet(G) | catB7-like, cmlA1-like | blaOXA-4, blaOXA-50-like, blaPAO-like, blaVIM-2 |  |  |
| 46 | aac(3)-Id-like, aadA2-like, aph(3')-IIb-like | fosA-like | sul1-like | dfrB5 | tet(G) | catB7-like, cmlA1-like | blaOXA-4, blaOXA-50-like, blaPAO-like, blaVIM-1, blaVIM-2 | aacA4-like | aac(6')Ib-cr-like |
| 47 | aac(3)-Id-like, aadA2-like, aph(3')-IIb-like | fosA-like | sul1 | dfrB5 | tet(G) | catB7-like, cmlA1-like | blaOXA-4, blaOXA-50-like, blaPAO-like, blaVIM-2 | aacA4-like | aac(6')Ib-cr-like |
| 48 | aac(3)-Id-like, aadA2-like, aph(3')-IIb-like | fosA-like | sul1-like | dfrB5 | tet(G) | catB7-like, cmlA1-like | blaOXA-4, blaOXA-50-like, blaPAO-like, blaVIM-1, blaVIM-2 | aacA4-like | aac(6')Ib-cr-like |
| 49 | aac(3)-Id-like, aadA2-like, aph(3')-IIb-like | fosA-like | sul1-like | dfrB5 | tet(G) | catB7-like, cmlA1-like | blaOXA-4, blaOXA-50-like, blaPAO-like | aacA4-like | aac(6')Ib-cr-like |
| 50 | aac(3)-Id-like, aadA2-like, aph(3')-IIb-like | fosA-like | sul1-like | dfrB5 | tet(G) | catB7-like, cmlA1-like | blaOXA-4, blaOXA-50-like, blaPAO-like, blaVIM-2 |  |  |
| 51 | aac(3)-Id-like, aadA2-like, aph(3')-IIb-like | fosA-like | sul1-like | dfrB5 | tet(G) | catB7-like, cmlA1-like | blaOXA-4, blaOXA-50-like, blaPAO-like, blaVIM-2 |  |  |
| 52 | aac(3)-Id-like, aadA2-like, aph(3')-IIb-like | fosA-like | sul1-like | dfrB5 | tet(G) | catB7-like, cmlA1-like | blaOXA-4, blaOXA-50-like, blaPAO-like, blaVIM-2 |  |  |
| 53 | aacA29-like | fosA-like | sul1 |  |  | catB7-like | blaOXA-50, blaPAO-like, blaVIM-2 |  |  |
| 54 | aacA29-like | fosA-like | sul1 |  |  | catB7-like | blaOXA-50, blaPAO-like, blaVIM-2 |  |  |
| 55 | aacA29-like | fosA-like | sul1 |  |  | catB7-like | blaOXA-50, blaPAO-like, blaVIM-2 |  |  |
| 57 | aac(3)-Id-like, aadA2-like, aph(3')-IIb-like | fosA-like | sul1 | dfrB5 | tet(G) | catB7-like, cmlA1-like | blaOXA-4, blaOXA-50-like, blaPAO-like, blaVIM-2 |  |  |
| 58 | aacA29-like, aadB, aph(3')-IIb-like | fosA-like | sul1 |  |  | catB7-like | blaOXA-10, blaOXA-50-like, blaPAO-like, blaVIM-2 |  |  |
| 59 | aacA29-like, aadB, aph(3')-IIb-like | fosA-like | sul1 |  |  | catB7-like | blaOXA-10, blaOXA-50-like, blaPAO-like, blaVIM-2 |  |  |
| 60 | aadB-like | fosA-like | sul1 |  |  | catB7-like | blaOXA-50, blaPAO-like | aacA4-like | aac(6')Ib-cr-like |
| 61 | aacA29-like, aadB, aph(3')-IIb-like | fosA-like | sul1 |  |  | catB7-like | blaOXA-10, blaOXA-50-like, blaPAO-like, blaVIM-2 |  |  |
| 62 |  | fosA-like | sul1 |  |  | catB7-like | blaOXA-50, blaPAO-like, blaVIM-2-like | aacA4-like | aac(6')Ib-cr-like |
| 63 | aadB | fosA-like | sul1-like |  |  | catB7-like | blaOXA-10, blaOXA-50, blaPAO-like, blaVIM-2-like | aacA4-like | aac(6')Ib-cr-like |
| 64 | aadB | fosA-like | sul1 |  |  | catB7-like | blaOXA-10, blaOXA-50, blaPAO-like | aacA4-like | aac(6')Ib-cr-like |
| 65 | aadB | fosA-like | sul1 |  |  | catB7-like | blaOXA-10, blaOXA-50, blaPAO-like | aacA4-like | aac(6')Ib-cr-like |
| 66 | aacA29-like, aadB | fosA-like | sul1 |  |  | catB7-like | blaOXA-10, blaOXA-50, blaPAO-like, blaVIM-2 | aacA4-like | aac(6')Ib-cr-like |
| 67 | aac(3)-Id-like, aadA2-like, aph(3')-IIb-like | fosA-like | sul1 | dfrB5 | tet(G) | catB7-like, cmlA1-like | blaOXA-4, blaOXA-50-like, blaPAO-like, blaVIM-2 |  |  |
| 68 | aac(3)-Id-like, aadA2-like, aph(3')-IIb-like | fosA-like | sul1 | dfrB5 | tet(G) | catB7-like, cmlA1-like | blaOXA-4, blaOXA-50-like, blaPAO-like, blaVIM-2 |  |  |
| 69 | aac(3)-Id-like, aadA2-like, aph(3')-IIb-like | fosA-like | sul1 | dfrB5 | tet(G) | catB7-like, cmlA1-like | blaOXA-4, blaOXA-50-like, blaPAO-like, blaVIM-2 |  |  |
| 71 | aac(3)-Id-like, aadA2-like, aph(3')-IIb-like | fosA-like | sul1 | dfrB5 | tet(G) | catB7-like, cmlA1-like | blaOXA-4, blaOXA-50-like, blaPAO-like, blaVIM-2 |  |  |
| 72 | aac(3)-Id-like, aadA2-like, aph(3')-IIb-like | fosA-like | sul1-like | dfrB5 | tet(G) | catB7-like, cmlA1-like | blaOXA-4, blaOXA-50-like, blaPAO-like, blaVIM-2 |  |  |
| 76 | aph(3')-IIb-like | fosA-like |  |  |  | catB7-like | blaOXA-50-like, blaPAO-like |  |  |
| 77 | aac(3)-Id-like, aadA2-like, aph(3')-IIb-like | fosA-like | sul1-like | dfrB5 | tet(G) | catB7-like, cmlA1-like | blaOXA-4, blaOXA-50-like, blaPAO-like, blaVIM-2 |  |  |
| 78 | aac(3)-Id-like, aadA2-like, aph(3')-IIb-like | fosA-like | sul1 | dfrB5 | tet(G) | catB7-like, cmlA1-like | blaOXA-4, blaOXA-50-like, blaPAO-like, blaVIM-2 |  |  |
| 79 | aac(3)-Id-like, aadA2-like, aph(3')-IIb-like | fosA-like | sul1-like | dfrB5 | tet(G) | catB7-like, cmlA1-like | blaOXA-4, blaOXA-50-like, blaPAO-like, blaVIM-2 |  |  |
| 80 | aac(3)-Id-like, aadA2-like, aph(3')-IIb-like | fosA-like | sul1-like | dfrB5 | tet(G) | catB7-like, cmlA1-like | blaOXA-4, blaOXA-50-like, blaPAO-like, blaVIM-2 |  |  |
| 81 | aac(3)-Id-like, aadA2-like, aph(3')-IIb-like | fosA-like | sul1 | dfrB5 | tet(G) | catB7-like, cmlA1-like | blaOXA-4, blaOXA-50-like, blaPAO-like, blaVIM-2 |  |  |
| 83 | aac(3)-Id-like, aadA2-like, aph(3')-IIb-like | fosA-like | sul1 | dfrB5 | tet(G) | catB7-like, cmlA1-like | blaOXA-4, blaOXA-50-like, blaPAO-like, blaVIM-2 |  |  |
| 85 | aac(3)-Id-like, aadA2-like, aph(3')-IIb-like | fosA-like | sul1-like | dfrB5 | tet(G) | catB7-like, cmlA1-like | blaOXA-4, blaOXA-50-like, blaPAO-like, blaVIM-2 |  |  |
| 86 | aac(3)-Id-like, aadA2-like, aph(3')-IIb-like | fosA-like | sul1 | dfrB5 | tet(G) | catB7-like, cmlA1-like | blaOXA-4, blaOXA-50-like, blaPAO-like, blaVIM-2 |  |  |
| 87 | aac(3)-Id-like, aadA2-like, aph(3')-IIb-like | fosA-like | sul1-like | dfrB5 | tet(G) | catB7-like, cmlA1-like | blaOXA-4, blaOXA-50-like, blaPAO-like, blaVIM-2 |  |  |
| 88 | aac(3)-Id-like, aadA2-like, aph(3')-IIb-like | fosA-like | sul1-like | dfrB5 | tet(G) | catB7-like, cmlA1-like | blaOXA-4, blaOXA-50-like, blaPAO-like, blaVIM-2 |  |  |
| 89 | aac(3)-Id-like, aadA2-like, aph(3')-IIb-like | fosA-like | sul1-like | dfrB5 | tet(G) | catB7-like, cmlA1-like | blaOXA-4, blaOXA-50-like, blaPAO-like, blaVIM-2 |  |  |
| 90 | aac(3)-Id-like, aadA2-like, aph(3')-IIb-like | fosA-like | sul1-like | dfrB5 | tet(G) | catB7-like, cmlA1-like | blaOXA-4, blaOXA-50-like, blaPAO-like, blaVIM-2 |  |  |
| 91 | aac(3)-Id-like, aadA2-like, aph(3')-IIb-like | fosA-like | sul1-like | dfrB5 | tet(G) | catB7-like, cmlA1-like | blaOXA-4, blaOXA-50-like, blaPAO-like, blaVIM-2 |  |  |
| 92 | aac(3)-Id-like, aadA2-like, aph(3')-IIb-like | fosA-like | sul1-like | dfrB5 | tet(G) | catB7-like, cmlA1-like | blaOXA-4, blaOXA-50-like, blaPAO-like, blaVIM-2 |  |  |
| 93 | aac(3)-Id-like, aadA2-like, aph(3')-IIb-like | fosA-like | sul1-like | dfrB5 | tet(G) | catB7-like, cmlA1-like | blaOXA-4, blaOXA-50-like, blaPAO-like, blaVIM-2 |  |  |
| 94 | aac(3)-Id-like, aadA2-like, aph(3')-IIb-like | fosA-like | sul1-like | dfrB5 | tet(G) | catB7-like, cmlA1-like | blaOXA-4, blaOXA-50-like, blaPAO-like, blaVIM-2 |  |  |
| 96 | aac(3)-Id-like, aadA2-like, aph(3')-IIb-like | fosA-like | sul1 | dfrB5 | tet(G) | catB7-like, cmlA1-like | blaOXA-4, blaOXA-50-like, blaPAO-like, blaVIM-2 |  |  |
| 97 | aac(3)-Id-like, aadA2-like, aph(3')-IIb-like | fosA-like | sul1-like | dfrB5 | tet(G) | catB7-like, cmlA1-like | blaOXA-4, blaOXA-50-like, blaPAO-like, blaVIM-2 |  |  |
| 98 | aac(3)-Id-like, aadA2-like, aph(3')-IIb-like | fosA-like | sul1-like | dfrB5 | tet(G) | catB7-like, cmlA1-like | blaOXA-4, blaOXA-50-like, blaPAO-like, blaVIM-2 |  |  |
| 99 | aac(3)-Id-like, aadA2-like, aph(3')-IIb-like | fosA-like | sul1 | dfrB5 | tet(G) | catB7-like, cmlA1-like | blaOXA-4, blaOXA-50-like, blaPAO-like, blaVIM-2 |  |  |
| 100 | aac(3)-Id-like, aph(3')-IIb-like | fosA-like | sul1-like | dfrB5 | tet(G) | catB7-like | blaOXA-50-like, blaPAO-like, blaVIM-2 |  |  |
| 101 | aac(3)-Id-like, aadA2-like, aph(3')-IIb-like | fosA-like | sul1-like | dfrB5 | tet(G) | catB7-like, cmlA1-like | blaOXA-4, blaOXA-50-like, blaPAO-like, blaVIM-2 |  |  |
| 102 | aac(3)-Id-like, aadA2-like, aph(3')-IIb-like | fosA-like | sul1-like | dfrB5 | tet(G) | catB7-like, cmlA1-like | blaOXA-4, blaOXA-50-like, blaPAO-like, blaVIM-2 |  |  |
| 103 | aac(3)-Id-like, aadA2-like, aph(3')-IIb-like | fosA-like | sul1-like | dfrB5 | tet(G) | catB7-like, cmlA1-like | blaOXA-4, blaOXA-50-like, blaPAO-like, blaVIM-2 |  |  |
| 104 | aac(3)-Id-like, aadA2-like, aph(3')-IIb-like | fosA-like | sul1 | dfrB5 | tet(G) | catB7-like, cmlA1-like | blaOXA-4, blaOXA-50-like, blaPAO-like, blaVIM-2 |  |  |
| 105 | aac(3)-Id-like, aadA2-like, aph(3')-IIb-like | fosA-like | sul1-like | dfrB5 | tet(G) | catB7-like, cmlA1-like | blaOXA-4, blaOXA-50-like, blaPAO-like, blaVIM-2 |  |  |
| 106 | aac(3)-Id-like, aadA2-like, aph(3')-IIb-like | fosA-like | sul1-like | dfrB5 | tet(G) | catB7-like, cmlA1-like | blaOXA-4, blaOXA-50-like, blaPAO-like, blaVIM-2 |  |  |
| 107 | aac(3)-Id-like, aadA2-like, aph(3')-IIb-like | fosA-like | sul1-like | dfrB5 | tet(G) | catB7-like, cmlA1-like | blaOXA-4, blaOXA-50-like, blaPAO-like, blaVIM-2 |  |  |
| 108 | aac(3)-Id-like, aadA2-like, aph(3')-IIb-like | fosA-like | sul1-like | dfrB5 | tet(G) | catB7-like, cmlA1-like | blaOXA-4, blaOXA-50-like, blaPAO-like, blaVIM-2 |  |  |
| 112 | aac(3)-Id-like, aadA2-like, aph(3')-IIb-like | fosA-like | sul1-like | dfrB5 | tet(G) | catB7-like, cmlA1-like | blaOXA-4, blaOXA-50-like, blaPAO-like, blaVIM-2 |  |  |
| 113 | aac(3)-Id-like, aadA2-like, aph(3')-IIb-like | fosA-like | sul1-like | dfrB5 | tet(G) | catB7-like, cmlA1-like | blaOXA-4, blaOXA-50-like, blaPAO-like, blaVIM-2 |  |  |
| 114 | aac(3)-Id-like, aadA2-like, aph(3')-IIb-like | fosA-like | sul1-like | dfrB5 | tet(G) | catB7-like, cmlA1-like | blaOXA-4, blaOXA-50-like, blaPAO-like, blaVIM-2 |  |  |
| 115 | aac(3)-Id-like, aadA2-like, aph(3')-IIb-like | fosA-like | sul1 | dfrB5 | tet(G) | catB7-like, cmlA1-like | blaOXA-4, blaOXA-50-like, blaPAO-like, blaVIM-2 |  |  |

**Supplementary Figure 1** Protein enrichment heatmap with gene presence (black) and absence (white) of the unique proteins in all strains pertaining to the *early*, *late* or *early and late* time group of cluster 2. Strain IDs are indicated horizontally and UniProt accession numbers are indicated vertically.

**a** Cluster 2 *early*.

**b** Cluster 2 *late*.

**c** Cluster 2 *early and late*.

This supplementary figure corresponds to Suppl. Tab. 6.

**Suppl. Fig. 1 a**

**Suppl. Fig. 1 b**

**Suppl. Fig. 1 c**
